# C1orf109L binding DHX9 promotes DNA damage depended on the R‐loop accumulation and enhances camptothecin chemosensitivity

**DOI:** 10.1111/cpr.12875

**Published:** 2020-08-06

**Authors:** Peng Dou, Yiqun Li, Haoxiu Sun, Wanqiu Xie, Xiaoqing Zhang, Xiaohan Zhang, Dandan Zhang, Shupei Qiao, Yanpeng Ci, Huan Nie, Fang Han, Yu Li

**Affiliations:** ^1^ School of Life Science and Technology Harbin Institute of Technology Harbin City China

**Keywords:** C1orf109L, chemotherapy, DNA damage, G2/M phase arrest, R‐loop, R‐loop‐associated proteins

## Abstract

**Objectives:**

R‐loop is a three‐stranded nucleic acid structure of RNA/DNA hybrid, which occurs naturally during transcription, and more R‐loop accumulation can trigger serious DNA damage. There has been increasing attention to the issue of R‐loop accumulation acted as a target for cancer therapy. However, the regulation of R‐loop‐associated proteins is poorly explored.

**Material and method:**

Quantitative real‐time PCR and Western blot were used to measure the expression of C1orf109 in cell lines. In addition, C1orf109L (C1orf109 longest isoform) protein binding partner was identified and validated using immunoprecipitation‐mass spectrometric (IP‐MS) and immunoprecipitation assays. DNA‐RNA immunoprecipitation (DR‐IP) and immunofluorescence determined the C1orf109L location on R‐loop. R‐loop accumulation was determined by immunofluorescence. Cell cycle was determined by flow cytometry. Finally, time‐lapse assay and cell counting were conducted to determined cell survival in response to camptothecin (CPT).

**Results:**

We found that C1orf109L could mediate cell cycle arrest in the G2/M phase and DNA damage depended on R‐loop accumulation. Meanwhile, C1orf109L could bind with DHX9 to trigger R‐loop accumulation. And C1orf109L was competitive with PARP1 binding to DHX9, which would block the function of DHX9‐PARP1 to prevent the R‐loop accumulation. Furthermore, C1orf109L could enhance the chemosensitivity of CPT, a chemotherapeutic drug capable of promoting R‐loop formation.

**Conclusions:**

Our data demonstrate that C1orf109L triggers R‐loop accumulation and DNA damage to arrest cell cycle.

## Graphical abstract

C1orf109L could compete with PARP1 for DHX9 binding, which mediates DNA damage and cell cycle arrest via triggering R‐loop accumulation. Furthermore, C1orf109L could enhance the chemosensitivity of camptothecin, a chemotherapeutic drug capable of promoting R‐loop formation.

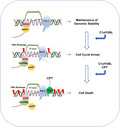

## 1 INTRODUCTION

A variety of endogenous or exogenous stimuli can induce DNA damage which could cause disease, including cancers and ageing. Serious DNA damage could directly lead to cell death. Hence, inducing DNA damage is an efficient way to control tumour growth. It has been proved that DNA damage could be induced by RNA/DNA hybrid structure, R‐loop.^1^ R‐loop is a three‐stranded nucleic acid structure formed during transcription, which comprises of nascent RNA hybridized with the DNA template, leaving the non‐template DNA single‐stranded.^2^ In several studies, R‐loop was revealed to play an essential biological role and involved in pathology. At physiological conditions, R‐loop could regulate immunoglobulin (Ig)G class switching in immune cells, DNA replication, DNA methylation and histone modifications.^3^, ^4^ In abnormal cells, R‐loops could promote DNA damage and induce cell cycle arrest, which was an important factor for genome instability, especially in cancer cells.^5^, ^6^ And deregulated R‐loop formation could result in aberrant transcriptional termination.^7^, ^8^ To date, R‐loop is considered as a target for cancer therapy, and many treatment drugs such as camptothecin (CPT) and topotecan can affect R‐loop formation.^9^

It has been found that R‐loop formation is regulated by R‐loop‐associated proteins, which is a same class of highly conserved RNA‐binding proteins (RBPs) in evolution.^10^ RBPs play a central role in the regulation of mRNA fate including the diversity and destiny of mRNAs and also are important players and coordinators in the maintenance of genome integrity and the modulation of R‐loop formation.^11^, ^12^ RBPs dysfunction could trigger R‐loop accumulation.^13^, ^14^ Wang IX et al reported that R‐loop‐related proteins involved in plenty of RBPs.^15^ In recent years, DHX9 helicase, a RNA‐binding protein, has been implicated in many fundamental cellular processes including DNA replication, transcription and genome stability. Meanwhile, DHX9 was required in the process of R‐loop formation; thus, it also was considered as an R‐loop‐associated protein.^16^ DHX9 interacts with PARP1, and both proteins are involved in regulation of R‐loop balance to prevent R‐loop‐associated DNA damage.^16^ And the deletion of DHX9 could promote R‐loop accumulation and enhance R‐loop‐induced DNA damage in response to an R‐loop enhancer, CPT.^16^, ^17^ Another study revealed that DHX9 was a key factor in the generation of R‐loops by RNA polymerase II and could interact with splicing factors to prevent R‐loop‐induced replication stress and genomic instability.^18^ When splicing factors SFPQ defected, DHX9 promoted R‐loop formation in cells by impairing RNA splicing. These studies indicated that DHX9 was an important factor to regulate R‐loop formation. And these researches also indicated that the regulation of R‐loop formation was a complex biological process which can be affected by different R‐loop‐associated proteins. R‐loop‐associated proteins are becoming a new hotspot, and the function of the proteins regulating R‐loop formation needs to be further explored.

*C1orf109* is a novel gene located on 1p34.3. The function of the gene has been poorly studied. Several research groups identified that C1orf109 dysregulation might involve in the developmental process or cause diseases such as tumours. Deletion of 1p34.3 locus, which includes *C1orf109*, could cause facial dysmorphism.^19^ The promoter region of *C1orf109* gene was modified by methylation in ageing and some diseases, such as keloids and systemic lupus erythematosus.^20^, ^21^, ^22^ Our group previously obtained C1orf109 shortest variant (203AA) from human lung tissue, and preliminary research discovered that this variant as a CK2 substrate involved in cell proliferation.^23^ However, very less was known about C1orf109 function because of multiple transcript variants of this gene existing. Here, we verified that C1orf109L, the longest variant of *C1orf109,* could trigger R‐loop accumulation and mediate DNA damage by competitive with PARP1 binding to DHX9. Furthermore, C1orf109L could be regarded as a therapeutic target in cancer treatment and it could be enhanced the chemosensitivity of CPT.

## 2 MATERIALS AND METHODS

### 2.1 Cell culture

HeLa, HEK‐293 and HEK‐293T cell lines were obtained from ATCC. The cells were cultured in DMEM containing 10% foetal bovine serum (Biological Industries, BI) and 1% penicillin‐streptomycin solution (Gibco) in a humidified incubator at 37°C with an atmosphere of 5% CO_2_.

### 2.2 Protein extraction and Western blotting

Proteins were extracted from subconfluent cultures of cells and then characterized by Western blot analysis. Cells were lysed in RAPI with phosphatase inhibitor cocktail, protease inhibitor cocktail, resolved on a sodium dodecyl sulphate‐polyacrylamide electrophoresis (SDS) gel and transferred onto a PVDF membrane (Millipore, Billerica, MA, USA). The membrane was blocked with 5% non‐fat milk in phosphate buffer saline (PBS) containing 0.05% Tween‐20 (PBST) for 1 hour at room temperature and then probed with a primary antibody overnight at 4°C. After extensive washing, the membrane was incubated with a secondary antibody conjugated to horseradish peroxidase (1:10 000, Proteintech) for 1 hour at room temperature. Blots were developed using ECL (Thermo Fisher Scientific, USA).

### 2.3 Flow cytometry analysis of cell cycle

Cells in different groups were trypsinized, washed once with PBS and fixed with 70% ethanol overnight at 4°C. After fixation, cells were washed once with PBS. After washing, cells were stained with PI/RNAse staining solution for 30 minutes (Tianjin Sungene Biotech, China). Flow cytometry (FCM) analysis was performed with a flow cytometer (BD Biosciences, USA).

### 2.4 Mass spectrometry analysis

Protein was added to a final concentration of 10 mmol/L dithiothreitol (DTT), followed by final concentration 55 mmol/L ammonium iodoacetate (IAM), and finally added 1 μg of Trypsin enzyme, overnight enzymatic hydrolysis 8‐16 hours. The enzymatically produced polypeptide was desalted by a C18 column, and the dehydrated polypeptide was dried and dissolved in 15 μL of Loading Buffer (0.1% formic acid, 3% acetonitrile). The peptide was analysed by LC‐MS/MS (ekspertTMnanoLC, AB Sciex TripleTOF 5600‐plus) instrument, and the results were evaluated.

### 2.5 Statistical analysis

All data were expressed in this manuscript as mean ± SD All the results have been performed at least three times by independent experiments. No samples and animals were excluded from the analysis. A two‐tailed Student *t* test was used to analyse the statistical significance between two groups. The statistical analysis was performed by using GraphPad prism 7.0 (GraphPad Software Inc). Asterisks indicate significant differences (*P < .05, **P < .01, ***P < .001).

For detailed experimental methods and materials, see Supplementary Materials and Table S7.

## 3 RESULTS

### 3.1 C1orf109L mediated proliferation inhibition of tumour cells via arresting cell cycle in G2/M phase

Although previous studies showed that C1orf109 dysregulation might cause diseases, its function was still unclear. NCBI database indicated that C1orf109 gene can produce multiple transcripts (Figure S1A), the gene encodes 4 transcripts with protein molecular weights of 280, 218, 265, 203 amino acids and predicted molecular masses of approximately 31.73KD, 24.77KD, 30.34KD and 23.38KD (http://www.novopro.cn/tools/protein). In this research, the function of C1orf109L protein was focused. First, we obtained the cell lines with lower expression of C1orf109L (Figure S1B), and analysed the cell viability of cancer cells and HEK‐293 cells by C1orf109L‐eGFP transient transfection. The exogenous expression of C1orf109L could obviously decrease the cell viability (Figure S1C). Next, the reliable doxycycline (DOX)‐inducible eGFP‐tagged C1orf109L Tet‐on HeLa cells were established (Figure S1D). The low expression of C1orf109L in various cells may be due to the presence of epigenetic regulation to inhibit its expression level. As Figure S1E shown, treating DNA methylation inhibitor (5‐azacytidine, 5‐aza) or histone acetylase inhibitor (Trichostatin A, TSA) to HeLa and detecting the expression level of C1orf109, it was found that TSA‐treated cells could significantly increase the RNA expression level of C1orf109. Detecting the level of C1orf109L protein after TSA treatment of cells, we found that with the extension of TSA treatment time, the increase of C1orf109L (280 amino acids) protein level be detected at a molecular weight of about 40KD (Figure S1F). As Figure S1G and H shown, the ability of colony formation and cell proliferation was reduced remarkably in HeLa cells with induced expression of C1orf109L (DOX+) (*P* < .001), comparison with the cells cultured in uninduced condition (DMSO, dimethylsulphoxide or DOX−).

**Figure 1:**
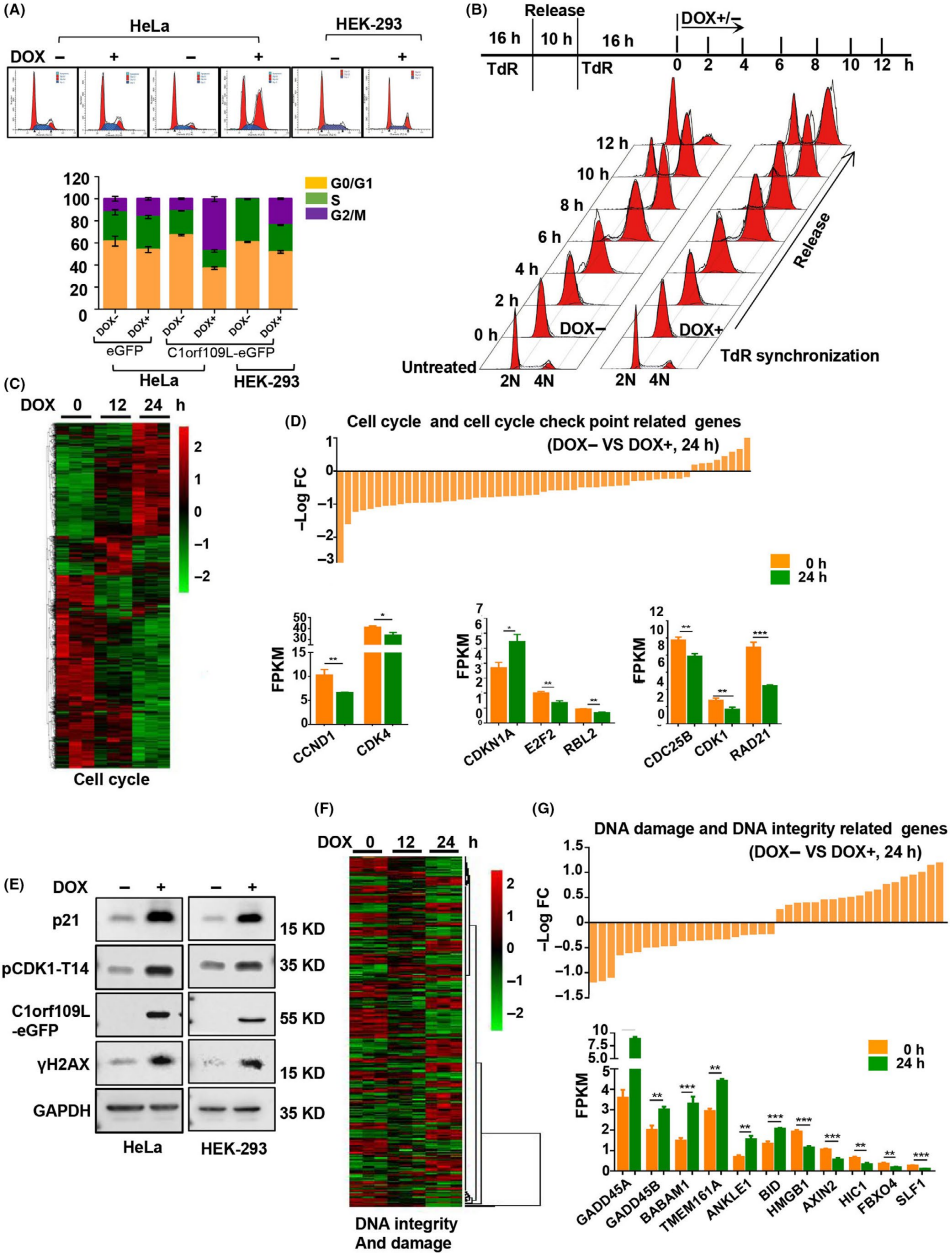
C1orf109L arrested cell cycle in G2/M phase and impaired gene expression. A, The exogenous expression of C1orf109L effected on the cell cycle progression in HeLa and HEK‐293 cells. Cells were either induced to express C1orf109L‐eGFP or not for 36 h. Cells were stained with propidium iodide (PI), and the cell cycle was analysed by flow cytometry. Data were presented as the mean ± SD based on three independent experiments. B, Tet‐on HeLa cells were synchronized at the G1/S boundary with TdR double blocking and then released with DOX induction over 12 h and collected at different time point along the determined cell cycle. C, Heat‐map of cell cycle‐related genes after inducing C1orf109L expression for 12 and 24 h. 0 h presented the control group that cell was not treated by DOX. D, Upper panel: The analysis of cell cycle and cell cycle check point‐related genes (DOX induced for 24 h). Data were presented as minus log fold change (–log FC). Lower panel: The expression level of G1/S phase and/or G2/M phase drivers was decreased, including CCND1, CDK4, CDK1, E2F2, DC25B and so on. Corresponding to this, the expression of CDKN1A was increased clearly. The –log FC and FPKM (fragments per kilobase million) data of RNA‐seq were showed by the mean ± SD based on three independent experiments. E, Western blot detected that the overexpression of C1orf109L‐eGFP over 36 h caused DNA damage to the signalling pathway, which included p21, pCDK1‐T14 and γH2AX in Tet‐on HeLa cells and Tet‐on HEK‐293 cells. F, Heat‐map for DNA integrity and damage‐related genes after induced C1orf109L expression for 12 and 24 h. 0 h presented the control group that cell was not treated by DOX. G, Upper panel: The analysis of DNA damage and DNA integrity‐related genes (DOX induced for 24 h). Data were presented as –log FC. Lower panel: The expression of GADD45A and B, BABAM1 and BID in response to DNA damage was increased prominently, while the expression of some genes that involved in DNA repair, such as AXIN2 and SLF1, was decreased. The –log FC and FPKM (Fragments Per Kilobase Million) data of RNA‐seq were showed by the mean ± SD based on three independent experiments

Moreover, the cell divisions of HeLa and HEK‐293cells were studied. As time lapse shown, the exogenous expression of C1orf109L (DOX+) could reduce undergoing cell division of HeLa cells (Video S1) compared with the control group (DOX−, Video S2). And then, cell cycle profile with C1orf109L expression was analysed (Figure 1A). HeLa and HEK‐293 cells with C1orf109L expression exhibited an abundant increase of cell population in the G2/M phase (*P* < .001). To further identify whether C1orf109L arrested the cell cycle at G2/M phase, Tet‐on HeLa cells were synchronized at the G1/S boundary with TdR, a drug that synchronizes the cell cycle, and then released by washing TdR and followed by culture with or without DOX‐induced C1orf109L expression for 12 hours. The results showed that the cell cycle was blocked from the G2/M (4N) to G1 (2N) phase at 10 hours after removing TdR (Figure 1B). Meanwhile, C1orf109L expression at the G1 phase induced by DOX impaired the transition from G1 phase to S phase (Figure S2).

### 3.2 The molecular basis of C1orf109L leading to cell cycle arrest

In order to dissect the molecular basis of C1orf109L‐mediating cell cycle arrest, the transcriptomes of HeLa cells with induced C1orf109L expression for 12 and 24 hours were analysed (Figure S3A), SRA accession number PRJNA558690. The remarkable changes of gene expression took place compared with cells untreated by DOX (Figure S3B). Using Gene Ontology (GO) annotations analysis, the gene expression profiles in cells of inducing C1orf109L compared with the cells without the induction were obtained (Figure S3C). The results showed that the expression of genes related to cell cycle checkpoint, mitotic cell cycle checkpoint and G1/S transition regulation was changed (Figure 1C and D upper panel, Tables S1 and S2). The expression level of G1/S phase and/or G2/M phase drivers was decreased, such as *CCND1*, *CDK4*, *CDK1*, *E2F2* and *CDC25B* (Figure 1D lower panel). Meanwhile, the expression of *CDKN1A*, a cell cycle checkpoint protein (p21) and inhibitor controlling the G1‐S and G2‐M phase,^24^, ^25^ clearly was increased (Figure 1D lower panel).

We further verified that p21 protein and phosphorylated CDK1 (pCDK1‐T14) were also markedly increased, when exogenous C1orf109L was expressed using DOX treated the HeLa and HEK‐293 cells for 24 hours (Figure 1E). The results of knocking down p21 with simultaneously induced C1orf109L expression indicated that C1orf109L could cause p21 up‐regulation (Figure S3D). And p21 silencing could significantly reverse the inhibition of cell proliferation in the DOX+ group (Figure S3E).

Considering p21 belongs to the downstream DNA damage pathway, we detected the expression of γH2AX protein, a DNA damage marker. The γH2AX was up‐regulated in both DOX+ groups of HeLa and HEK‐293 cells (Figure 1E). Additionally, it is worthy to note that differential transcriptome data of HeLa cells of inducing C1orf109L expression were involved in signalling pathways of DNA integrity and DNA damage, and cell death (Figure 1F and Figure S3C, Tables S3 and S4). Specially, the expression of genes in response to DNA damage was increased prominently, such as *GADD45A* and *B*, *BABAM1* and *BID*, while the expression of some genes that involved in DNA repair was decreased (Figure 1G). These data suggested that induced C1orf109L expression could lead to DNA damage, and the cell cycle arrest may be the response of cells to DNA damage.

### 3.3 C1orf109L interaction network and binding protein analysis

**Figure 2:**
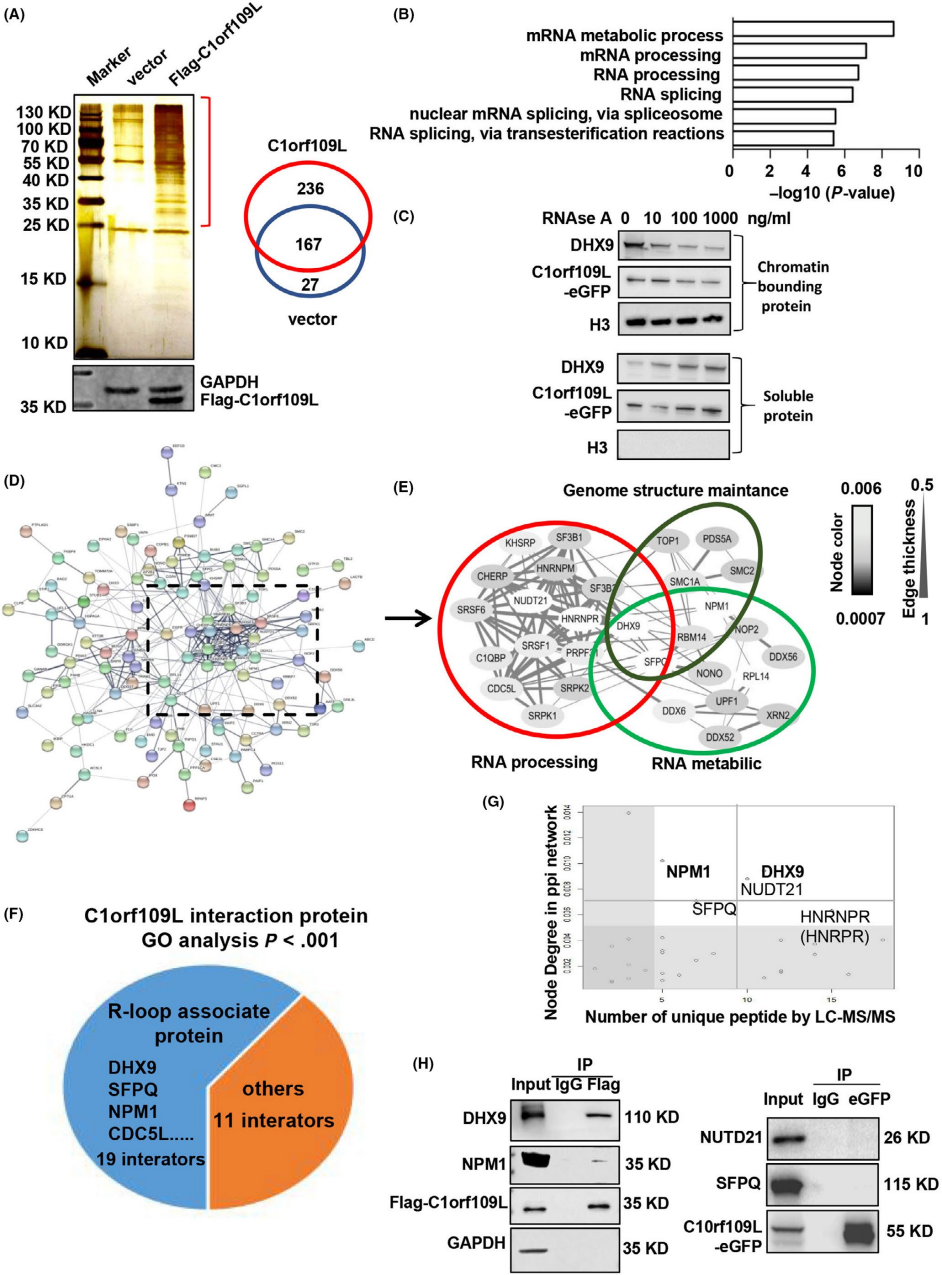
C1orf109L interaction network and binding protein analysis by IP‐MS. A, Left: The proteins from whole‐cell lysates (WCLs) were immunoprecipitated using Flag‐M2 beans, after Flag‐C1orf109L or Flag alone were transfected into HeLa cells. Immunoprecipitated proteins were analysed by SDS‐PAGE and silver staining, and the gel pieces containing regions of interest were analysed by LC‐MS/MS identify proteins immunoprecipitated with Flag‐C1orf109L. Right: Venn diagram showed the number of C1orf109L interactors. B, GO analysis of the C1orf109L‐interaction protein. C, C1orf109L binding chromatin was dependent on RNA. HeLa cell lysates were incubated with indicated amounts of RNase A for 20 min on ice before separation of the chromatin‐bound and soluble fractions. The amount of chromatin‐bound C1orf109L‐eGFP and DHX9 in the presence of RNase A was examined by Western blot. D, Curated protein‐protein interactions (PPI) among identified C1orf109L binding partners are represented in a PPI network. E, Select the dense part in the PPI network, edge thickness indicates the confidence score for the interaction, and node colour indicates the abundance of the interactors in the Flag‐C1orf109L immunoprecipitation. Biological functions of the identified protein complexes are indicated in the coloured Venn diagram superimposed on the network. F, Analysis of the interaction protein known to bind an RNA/DNA hybrid in the C1orf109L interaction proteome (GO analysis, *P* < .001). G, C1orf109L interactors were prioritized based on their degree of interconnection and the number of unique peptides/amino acid length identified by MS. In the scatterplot, node degree in the PPI network (y‐axis) identifies hubs in the Flag‐C1orf109L PPI network, while the number of unique peptides (x‐axis) reflects the abundance of the indicated protein in the purified Flag‐C1orf109L protein complex. H, Validation of the C1orf109L interactor that was co‐immunoprecipitated with Flag‐C1orf109L and C1orf109L‐eGFP in HeLa cells

To clarify the binding target of C1orf109L, C1orf109L‐interacting proteins were detected by tandem mass spectrometry‐based affinity proteomics, using Flag‐tagged C1orf109L immunoprecipitated from HeLa cells at 24 hours after transfection. There are 236 proteins specifically interacted with C1orf109L (Figure 2A and Table S5), of which the functions are mainly rich in RNA metabolism and processing, as determined through GO annotations (Figure 2B). To verify a protein which binding to RNA or RNA‐binding proteins (RBPs),^26^ RNase A was utilized to digest RNA of chromatin. As similar trend as positive control of RNA‐binding protein DHX9, chromatin‐associated C1orf109L protein level was reduced by the treatment of RNase A in a dose‐dependent manner in HeLa cells (Figure 2C). The results indicated that C1orf109L could bind RNA or RNA‐binding proteins.

Furthermore, the multiple sets of C1orf109L‐interacting proteins were showed by Enrichment analysis based on GO annotations (Figure 2D). The region of high edge thickness and coverage rate included 30 proteins related to RNA processing, RNA metabolism and genome structure maintenance (Figure 2E and Table 1). Notably, nineteen of them were R‐loop‐associated proteins (Figure 2F and Table S6), comparison with reported data (PXD002960 in Table S6).^16^ The evaluation of the node degree in the PPi network showed significant unique peptide numbers of some R‐loop proteins, included DHX9, NPM1, NUDT21, SFPQ and HNRNPR (Figure 2G). Thus, immunoprecipitation studies were performed to further confirm interaction between the C1orf109L and the other proteins. The results indicated that C1orf109L could interact with DHX9 and NPM1 (Figure 2H), which were R‐loops‐associated proteins and involved in RNA processing and RNA metabolism.^27^, ^28^

**Table 1:** C1orf109L interacted with proteins of RNA processing, RNA metabolic and Genome structure maintenance

	Protein	Description	Fold enrichment (Hybrid/dsDNA)	
			Unused	Unique Peptide
1	DHX9	ATP‐dependent RNA helicase A	24.58	12
2	SFPQ	Splicing factor, proline‐ and glutamine‐rich	8.57	5
3	SMC1A	Structural maintenance of chromosomes protein 1A	8.32	4
4	CDC5L	Cell division cycle 5‐like protein	6.61	3
5	HNRNPR(HNRPR)	Heterogeneous nuclear ribonucleoprotein R	5.92	4
6	NPM1(NPM)	Nucleophosmin	5.40	3
7	KHSRP(FUBP2)	KH‐type splicing regulatory protein	4.66	3
8	RPL14(RL14)	Ribosomal protein L14	4.18	3
9	DDX6	Probable ATP‐dependent RNA helicase DDX6	4.00	2
10	PRPF31(PRP31)	U4/U6 small nuclear ribonucleoprotein Prp31	4.00	2
11	SRPK1	SRSF protein kinase 1	4.00	2
12	SF3B1	Splicing factor 3b subunit 1	3.6	2
13	RBM14	RNA‐binding protein 14	3.43	2
14	HNRNPM(HNRPM)	Heterogeneous nuclear ribonucleoprotein M	3.09	1
15	NOP2	Probable 28S rRNA (cytosine(4447)‐C(5))‐methyltransferase	3.02	2
16	NUDT21(CPSF5)	Nudix hydrolase 21	2.88	2
17	SRSF1	Serine/arginine‐rich splicing factor 1	2.13	1
18	TOP1	DNA topoisomerase I	2.11	1
19	DDX56	DEAD‐box helicase 56	2.10	1
20	SF3B3	Splicing factor 3b subunit 3	2.02	2
21	XRN2	5‐3 exoribonuclease 2	2.01	1
22	CHERP	Calcium homeostasis endoplasmic reticulum protein	2.00	1
23	NONO	Non‐POU domain‐containing octamer‐binding protein	2.00	1
24	C1QBP	Complement C1q binding protein	2.00	1
25	SRPK2	SRSF protein kinase 2	2.00	1
26	PDS5A	PDS5 cohesin‐associated factor A	2.00	1
27	SMC2	Structural maintenance of chromosomes protein 2	1.85	1
28	DDX52	DExD‐box helicase 52	1.77	1
29	UPF1(RENT1)	Regulator of nonsense transcripts 1	1.76	1
30	SRSF6	Serine and arginine‐rich splicing factor 6	1.51	1

### 3.4 C1orf109L binding with DHX9 disturbs the interaction between DHX9 and PARP1

**Figure 3:**
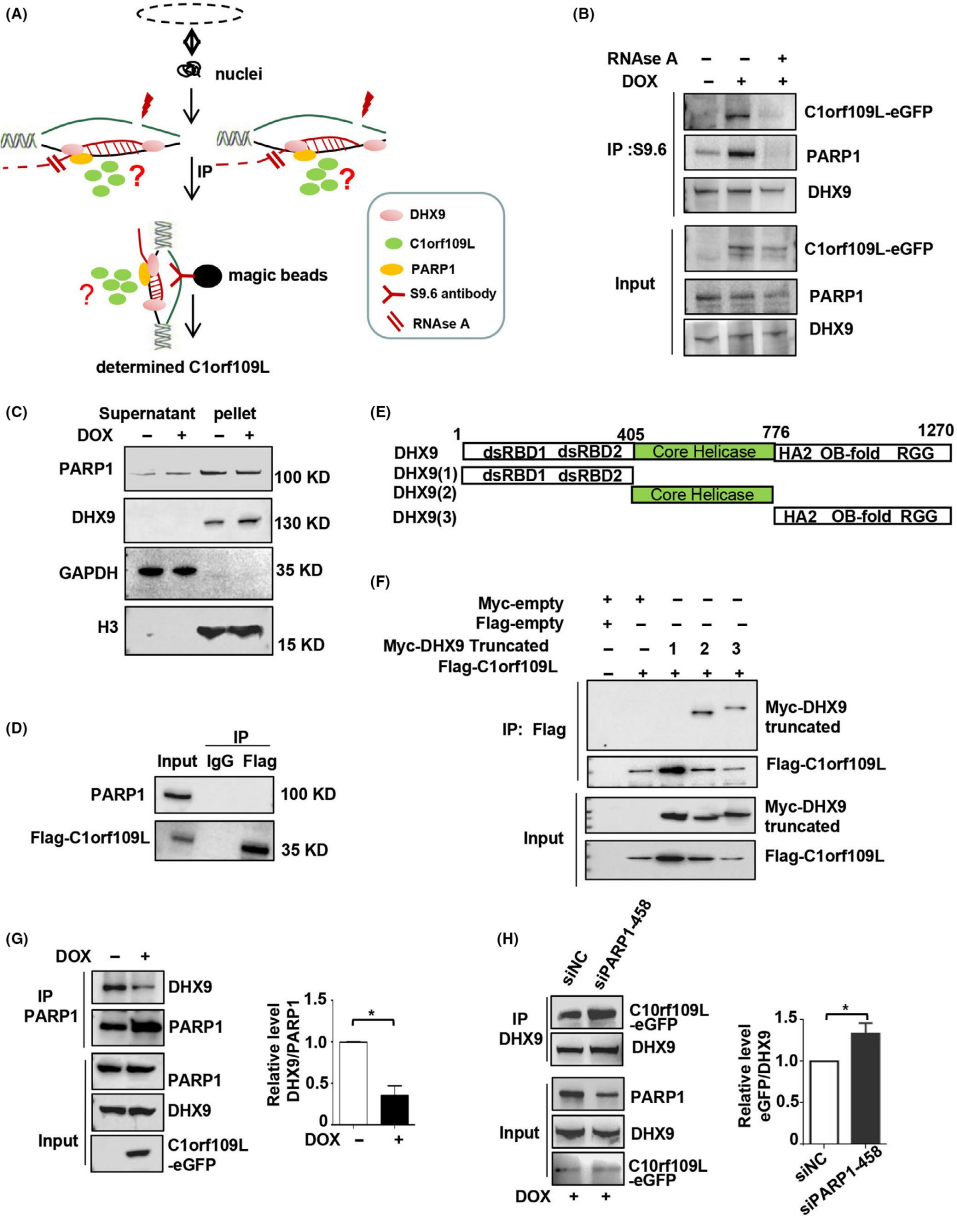
C1orf109L bound with DHX9 and disturbed the interaction between DHX9 and PARP1. A, Working model for DR‐IP, induced C1orf109L‐eGFP expression for 24 h in HeLa cells; isolated cell nuclei and a working model for separated R‐loop and ultrasonication. The supernatant was treated with or without RNAse A for 20 min, and the immunoprecipitated R‐loop was treated with S9.6 and determined with a GFP antibody. B, The R‐loop‐associated proteins were obtained from isolated HeLa cell nuclei, using Immunoprecipitate of S9.6 antibody. C1orf109L, DHX9 and PARP1 of the protein complex was detected by Co‐IP in C1orf109L expression cells with RNase A treatment or RNase A untreatment, the control group was without RNase A and DOX (DMSO treated). C, Western blot tested the PARP1and DHX9 in supernatant and pellet of isolated HeLa cell nuclei. The results indicated that the expression of C1orf109L did not affect the localization of DHX9 and PARP1 on chromatin RNA. D, IP using Flag antibody, the data determined that the PARP1 was not the interactor of the C1orf109L. E to F, The model of different domain of DHX9 (E) and C1orf109L binding with core helicase domain and C‐terminal of DHX9 (F). Myc‐tag truncates of DHX9 and Flag‐C1orf109L were cotransfected into HeLa cells. Lysates cells and immunoprecipitated with Flag‐M2 beans and determined DHX9 truncation with Myc antibody. G to H, The lysates cells of Tet‐on HeLa cells with C1orf109L was immunoprecipitated by PARP1 antibody, and then, DHX9 and PARP1 were detected by Western blot. The data showed that the C1orf109L expression could reduce PARP1 binding to DHX9, *P < .05 (G). After PARP1 knocking down in Tet‐on HeLa cells with C1orf109L expression, the ability of C1orf109L binding with DHX9 was increased, *P < .05 (H)

DHX9 and PARP1 play an important role in regulating R‐loop turn‐over.^16^ Therefore, the DNA‐RNA immunoprecipitation (DR‐IP) experiments were performed to detect the R‐loop‐associated protein (Figure 3A). R‐loops were extracted by an R‐loop‐specific antibody, S9.6, from isolated HeLa cell nuclei. The results showed that, similar to PARP1, C1orf109L in cells with RNase A treatment was markedly reduced compared with the control group without RNase A (Figure 3B). However, the expression of C1orf109L did not affect the localization of DHX9 and PARP1 on chromatin RNA (Figure 3C), and C1orf109L did not bind with PARP1 (Figure 3D).

To verify the relation between the C1orf109L and DHX9, the three DHX9 truncates were constructed (Figure 3E), including the double‐strand RNA‐binding domain 1 and 2 (dsRBD1, ds RBD2), core helicase domain and C‐terminus of DHX9 with repeated arginine and glycine‐glycine (RGG) regions.^29^, ^30^, ^31^ The Co‐IP results showed that C1orf109L could bind with core helicase domain and C‐terminal of DHX9 (Figure 3F). C1orf109L binding chromatin dependent on RNA further confirmed that C1orf109L shared the same region with DHX9 on chromatin RNA (Figure 2C). Moreover, the data using PARP1 antibody immunoprecipitation revealed that C1orf109L high expression could reduce PARP1 binding to DHX9 (Figure 3G). Meanwhile, when PARP1 was knocking down, the amount of DHX9‐bound C1orf109L‐eGFP was increased (Figure S4B and Figure 3H). These results suggested that C1orf109L might bind to DHX9 competing with PARP1.

### 3.5 C1orf109L binding DHX9 triggered R‐loop accumulation and mediated DNA damage

**Figure 4:**
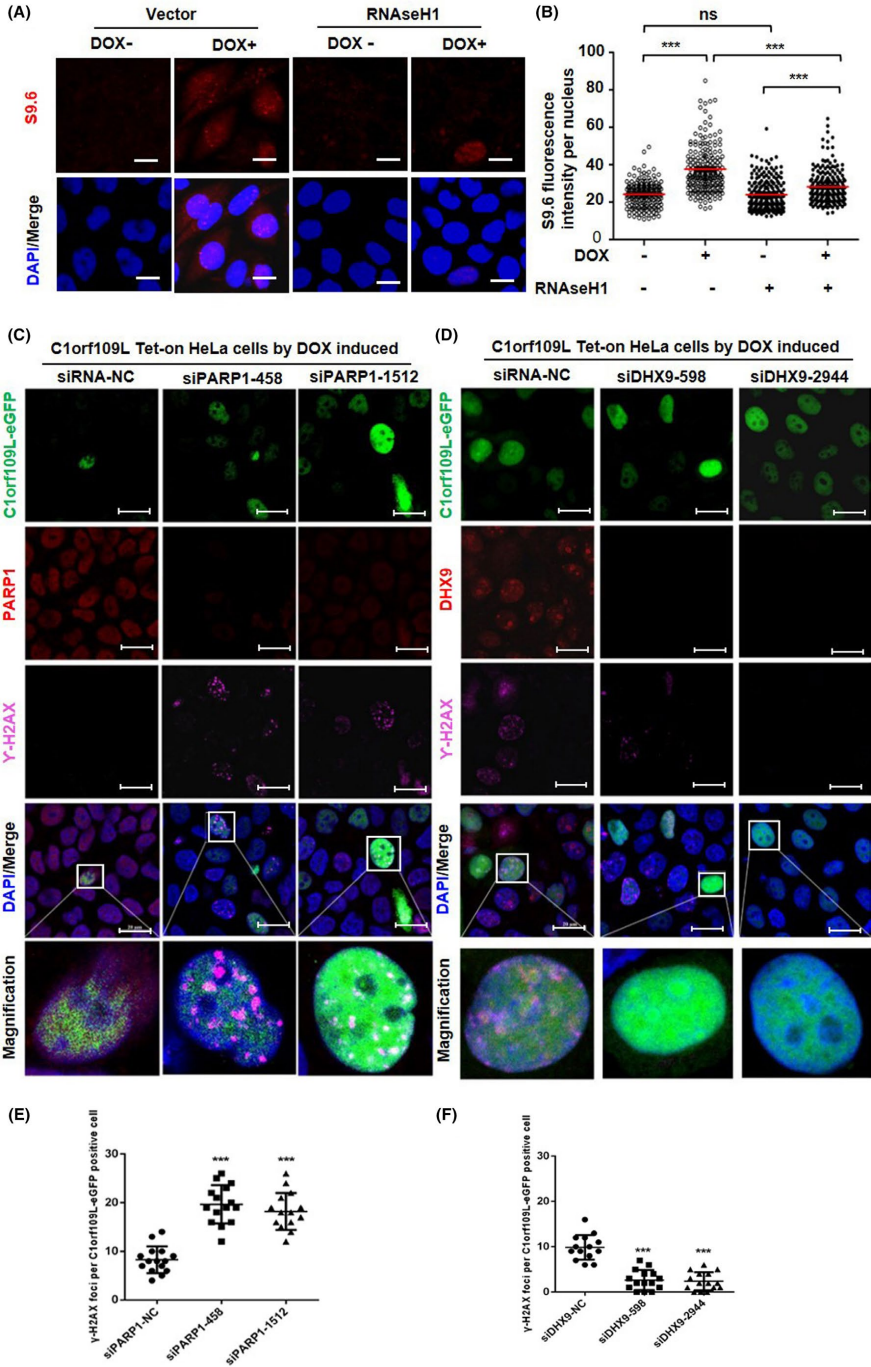
C1orf109L trigger DNA damage depending on R‐loop accumulation. A, Immunofluorescence analysed the R‐loop accumulation in C1orf109L‐eGFP Tet‐on HeLa cells, after the cells were induced by DOX 24 h, with or without the overexpression of RNaseH1‐eGFP. The R‐loop‐specific S9.6 antibody (red) and DAPI (blue) were used. Representative images bars: 20 µm. B, S9.6 fluorescence intensity (R‐loop) per nucleus from a representative experiment (≥200 nuclei were analysed by image J). The horizontal red bars represent the means, and each dot is one nucleus. ***P < .001; ns, not from a representative experiment (≥200 nuclei were analysed). The horizontal red bars represent the means, and each dot is one nucleus. ***P < .001, ns, not significant (Student's *t* test). C, Immunofluorescence of γH2AX analysed in C1orf109L Tet‐on HeLa cells with PARP1 knocking down. Green: C1orf109L‐eGFP, Red: PARP1, Purple: γH2AX, Blue: DAPI. Bars: 20 µm. D, Immunofluorescence of γH2AX analysed in C1orf109L Tet‐on HeLa cells with DHX9 knocking down. Green: C1orf109L‐eGFP, Red: DHX9, Purple: γH2AX, Blue: DAPI. Bars: 20 µm. E to F, Quantification was average of three independent experiments, and 5 cells were counted for each experiment. Data are presented as mean ± SD ***P < .001

The relationship between C1orf109L and R‐loop was further analysed because the DHX9 could promote R‐loop formation but DHX9 interacting with PARP1 could prevent R‐loop‐associated DNA damage.^16^, ^17^, ^18^ The R‐loop in cells with exogenous expression of C1orf109L was detected by immunofluorescence, using S9.6 antibody. Meanwhile, RNaseH1, an R‐loop digestion enzyme, was overexpressed to explore the function of C1orf109L to regulate R‐loop formation. The results exhibited that the fluorescence intensity of S9.6 was remarkably increased in the cell nuclei with DOX treatment (*P* < .001). But when R‐loops were digested, the fluorescence intensity was obviously decreased in the cell nuclei with DOX+ (*P* < .001) **(**Figure 4A and B**)**.

Moreover, when PARP1 was knocked down (Figure S4A), the fluorescence intensity of γH2AX was significantly increased in C1orf109L with DOX treatment **(**Figure 4C and E**)** while was decreased in cells **(**Figure 4D and F**)** with C1orf109L expression and DHX9 knocking down **(**Figure S4B**)**. And S9.6 fluorescence appeared to reduce in cell with C1orf109L expression and DHX9 knocking down (Figure S4C and D), whereas the opposite result was found in cells with C1orf109L expression and PARP1 knocked down (Figure S4E and F). These data furthermore demonstrated that C1orf109L might trigger R‐loop accumulation by competing with PARP1 to bind with DHX9, and C1orf109L was dependent on DHX9 to mediated DNA damage.

In addition, we proved the inhibition of cell proliferation by C1orf109L promoting R‐loop information. As shown in Figure [Link], [Link]A and B, the colony number of cells was clearly reduced with exogenous expression of C1orf109L and RNAseH1‐eGFP expression. And then, RNAseH1‐eGFP overexpression in Tet‐on HeLa cells could reverse the phenotype which the C1orf109L leading to the inhibition of cell proliferation (Figure S5C). The results revealed that the role of C1orf109L inhibiting cell proliferation was depended on R‐loop accumulation.

### 3.6 C1orf109L induced enormous DNA damage by promoting R‐loop accumulation in response to CPT

**Figure 5:**
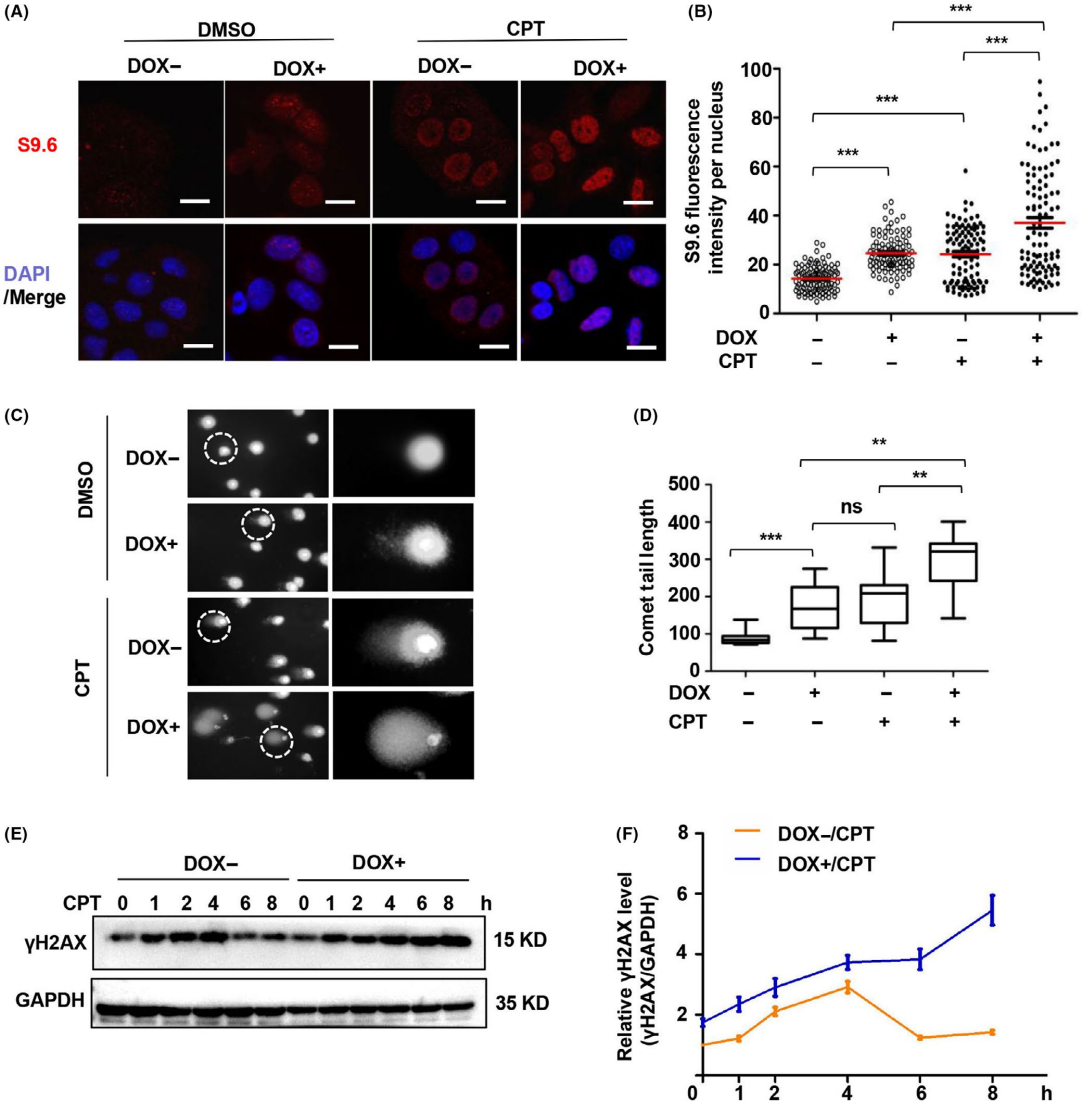
C1orf109L promote DNA damage in response to CPT. A, Immunofluorescence analysed in C1orf109L‐eGFP Tet‐on HeLa cells with DOX for 36 h and with 10 µmol/L CPT treatment for 4 h, the control cells were treated with DMSO. The R‐loop‐specific S9.6 antibody (red) and DAPI (blue) were used. Image bars: 20 μm. B, R‐loop fluorescence intensity per nucleus from a representative experiment (≥100 nuclei were analysed by image J). The horizontal red bars represent the means, and each dot is one nucleus. ***P < .001, as based on Student's *t* test. C to D, Comet assay was used to analyse the DNA damage of Tet‐on HeLa cells, which either did or did not induce C1orf109L‐eGFP expression by DOX or DMSO for 36 h, and another group cells were treated with 10 µmol/L CPT for 4 h. The zoomed‐in picture showed the nucleus circled by a white frame (C). The comet movement tail was calculated (cell number: ≥30). The results were presented as the mean ± SD (E). E to F, Western blot detected the γH2AX protein levels of Tet‐on HeLa cells, which were treated with or without DOX under CPT treatment for 0, 1, 2, 4, 6 and 8 h (E). Analysis of the relative γH2AX protein level (γH2AX/GAPDH) using Image J (F)

As noted in the transcriptome data analysis, C1orf109L could mediate the expression of genes regulating DNA integrity, DNA damage and cell death. For this reason, camptothecin (CPT), a widely used as a R‐loop activator,^32^ was introduced into our study. Interestingly, the nuclear S9.6 fluorescence intensity in C1orf109L expression cells was remarkably increased (*P* < .001) in response to CPT treatment (Figure 5A and B). And we further visualized colocalization of C1orf109L‐eGFP (green) and the R‐loops (red, stained by the S9.6 antibody) in nuclei of C1orf109L‐eGFP cell with CPT treatment. As shown in a large‐scale image of Figure S6, the colocalization for C1orf109L and R‐loops was further pronounced after CPT treatment, while the yellow fluorescence was rather weak in C1orf109L‐eGFP cell with DMSO treatment.

R‐loop accumulation is an important reason for DNA damage. To identify whether C1orf109L with CPT treatment could trigger enormous DNA damage, a comet assay was conducted. The results showed that the comet tail of the DOX+ group was remarkably longer than that of the DOX− group, especially DOX+ group with CPT treatment (Figure 5C and D). Subsequently, Tet‐on HeLa cells were treated with CPT for the indicated time, as shown in Figure 5E and F, with or without DOX for 24 hours, and γH2AX was detected by Western blotting. The results indicated that the extra expression of γH2AX in the DOX+ group could last over 8 hours, while in DOX− group it was only 4 hours under CPT treatment (Figure 5E and F).

The data demonstrated that C1orf109L could cause serious R‐loop‐associated DNA damage and promote γH2AX up‐regulation in a time‐dependent manner in response to CPT.

### 3.7 C1orf109L enhanced chemosensitivity of CPT

**Figure 6:**
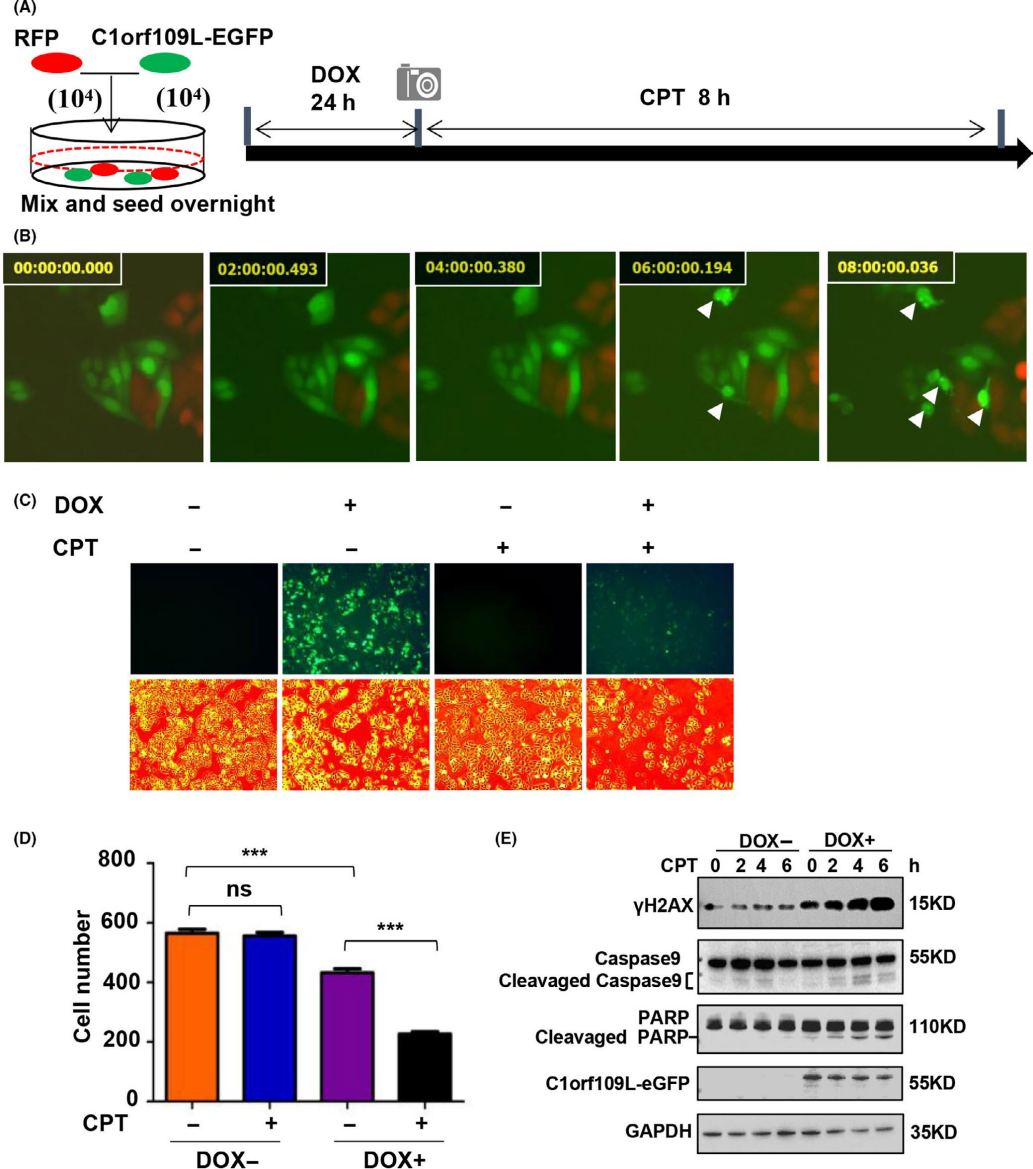
C1orf109L activated DNA damage and apoptosis pathways and enhanced CPT sensitivity. A to B, The same number of stable transfection Tet‐on HeLa cells expressing RFP and C1orf109L‐eGFP, respectively, were seeded on a plate overnight and induced with DOX for 24 h. Treatment with 10 μmol/L CPT and using time‐lapse photography (A). Time lapse showing the cell phenotype in response to CPT treatment (B, Video S3). White arrows presented the pyknosis cells. C to D, DOX‐induced Tet‐on HeLa cells to express C1orf109L‐eGFP within 24 h. The cells were photographed following treatment with DMSO or 10 µmol/L of CPT for 12 h, representative images bars: 200 µm. C, Calculated cell number (D). The result was presented as the mean ± SD for five views. **P < .01, ns, not significant. Based on Student's *t* test. E, C1orf109L‐eGFP expression was induced in Tet‐on HeLa cells for 36 h; cells were treated with 10 µmol/L of CPT for 1, 2, 4, and 6 h, respectively. Western blot analysis of the effect of the cell death pathway, including caspase9 and PARP1

Based on the above findings, a time‐lapse system was designed to record the phenotype of C1orf109L expression in response to CPT. With the HeLa cells harbouring DOX‐induced RFP as a control (red cells), DOX‐induced C1orf109L‐eGFP HeLa cells (green cells) were seeded on a plate and cultured in the presence of DOX for 24 hours and recorded the cell every 10 mins by time lapse at 488nm and 568nm wave length over 8 hours. As shown in Figure 6A, the green cells (expressing C1orf109L‐eGFP) began to die after about 5 hours of CPT treatment, and the red cells (RFP expression) remained alive until 8 hours (Figure 6B and Video S3).

To further clarify that C1orf109L could promote cell death with CPT treatment, we performed HeLa cell in response to CPT for 12 hours, and the cell numbers were calculated. The results showed that the cell number was no difference between the CPT+/DOX− group and CPT‐/DOX− group. However, the number of cells in the CPT+/DOX+ group had remarkably reduced compared with that in the CPT‐/DOX+ group (Figure 6C and D). Western blot analysis also indicated that under C1orf109L expression and CPT treatment, caspase‐9, up‐stream of caspase‐3, was activated and PARP1, substrate of caspase‐3, was cleaved (Figure 6E). The results verified that the cell death induced by C1orf109L was in the manner of cell apoptosis in cells with CPT treatment and that C1orf109L could enhance cellular chemosensitivity to CPT.

**Figure 7:**
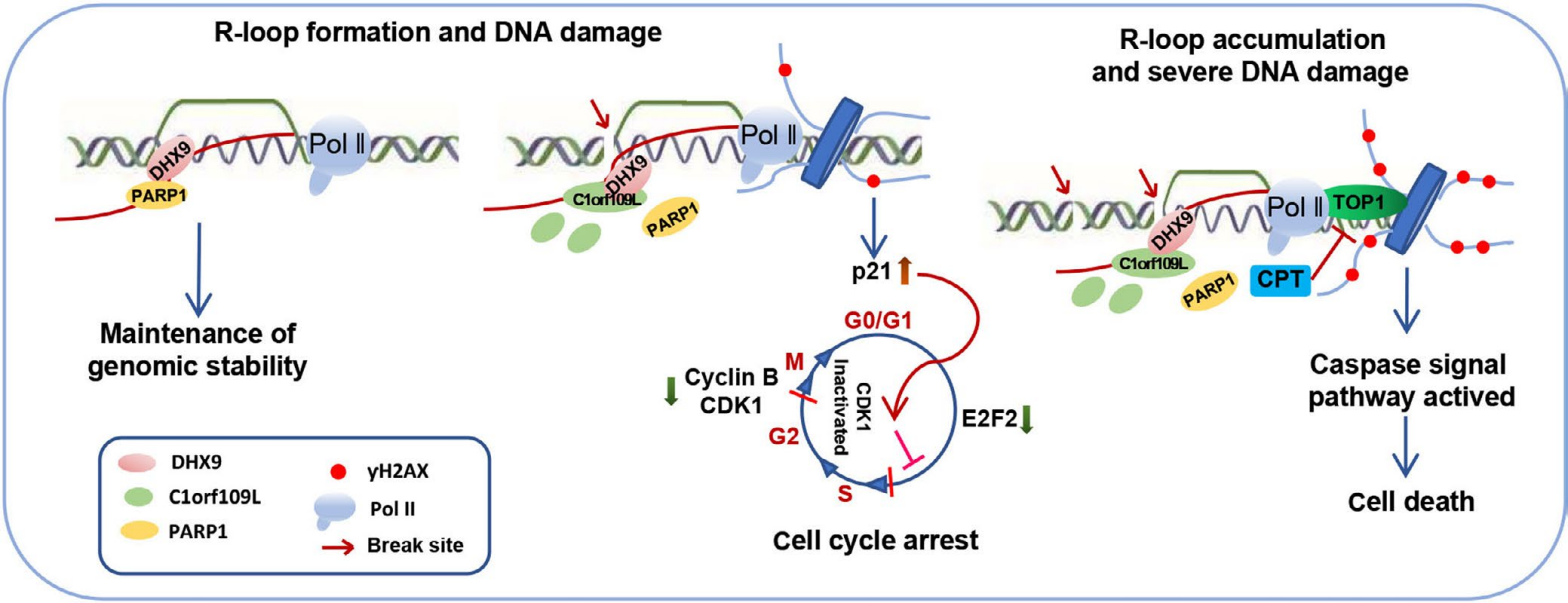
The working model of C1orf109L binding DHX9 promotes DNA damage depended on the R‐loop accumulation

Hence, the working model of C1orf109L triggering R‐loop accumulation and enhancing CPT chemosensitivity was drawn (Figure 7). C1orf109L could disturb the balance of R‐loop by competing with PARP1 and block the function of PARP1‐DHX9 which maintain a balance of R‐loop. It could mediate cell cycle arrest and DNA damage by promoting R‐loop formation and enhance the chemosensitivity of CPT by R‐loop excessive accumulation inducing enormous DNA damage.

## 4 DISCUSSION

Although a previous study reported that C1orf109 expression was regulated by hyper‐methylation of its promoter,^21^ function is largely uncovered. And C1orf109 could transcript multiple isoforms. Hence, its function might be more complicated yet remains unclear. Our previous investigation disclosed that the shortest variants of *C1orf109* as a CK2 substrate involved in cell proliferation.^23^ Here, we tested the expression levels of C1orf109 and discovered that low level of C1orf109S expression in various cells, however, could not detect the expression of C1orf109L protein in these cells. The reason is probably that the gene has abnormal epigenetic regulation in immortalized cells, and previous studies had discovered that promoter of this gene is hypermethylated in keloids.^21^ However, we found that there is a molecular weight gap of about 3KD between the endogenous C1orf109L and the overexpressed Flag‐C1orf109L. The reason may be that there is some modification of the endogenous C1orf109L induced by TSA that leads to an increase in molecular weight. C1orf109S protein level not be affected with TSA treatment, the main reason is that the expression level of C1orf109 different transcripts may be regulated by different transcription factors, and regulated transcription factors of C1orf109L may be more significantly affected by TSA. We demonstrated that the exogenous expression of C1orf109L could inhibit cell viability by arresting cell cycle in G2/M phase. And we found that C1orf109L expression caused clearly changes of DNA damage‐related genes and DNA integrity‐associated genes. Further analysis revealed that C1orf109L promoted DNA damage and activated DNA damage signalling pathway, which seen by increasing p21 protein level and phosphorylation‐mediated inactivation of CDK1, a G2 to M phase control protein.^23^ And C1orf109L transient expression could induce the DNA damage and the expression level of *GADD45* and γH2AX was increased evidently.

We identified the C1orf109L binding proteins by IP‐MS and Western blotting to analyse the mechanism of C1orf109L‐mediated DNA damage and attempted to explore the function of C1of109L. Our results indicated that C1orf109L could bind to DHX9, an important R‐loop‐associated protein, which also participates in pre‐mRNA processing process, and enhance DNA damage of dependent on R‐loops. It has been reported that the dysfunction of RNA processing protein could inhibit cell proliferation via forming R‐loops.^33^, ^34^ In physiological processes, R‐loops is a key structure of transcription regulation, and some RNA‐binding proteins regulate R‐loop balance.^16^ Nevertheless, once the balance is broken, R‐loop excessive accumulation would cause cancers and neurodegenerative diseases.^7^, ^35^

Usually, R‐loops are very rare in cells and exist in a dynamic way. But its excessive accumulation is harmful to cells. R‐loop accumulation could induce the genome instability and activate the cell cycle checkpoint, which is one of the most notable mechanisms leading to growth inhibition.^36^ RNAse A can be used to remove the single‐stranded RNA (ssRNA), which is the ssRNA on the outside of the R‐loop and is not complementary to the DNA.^16^ For the R‐loop binding protein DHX9, on the one hand, DHX9 can bind to the R‐loop with unwinding activity. On the other hand, DHX9 can bind to the ssRNA on the outside of the R‐loop and participate in the regulation and affect the RNA alternative splicing to promote R‐Loop formation.^18^ DHX9 bind to PARP1 on ssRNA, so PARP1 will be removed from R‐loop outside of ssRNA with RNAse A treatment.^16^ In our work, proteins interacting with C1orf109L were also involved in R‐loop regulation. Our data exhibited that C1orf109L colocalized with the R‐loops and bound to DHX9 by competitive with PARP1 at the RNA‐DNA hybrid region on chromatin, which was verified by DR‐IP with RNase A treatment and co‐immunoprecipitation. Moreover, the expression of C1orf109L could trigger R‐loop accumulation.

Although some R‐loop‐associated proteins were identified in cells,^15^, ^16^, ^37^ the regulation of R‐loop formation remains elusive. In this study, our data clarified that C1orf109L could interact with DHX9, an RNA helicase catalysing the ATP‐dependent unwinding of double‐stranded RNA and DNA‐RNA complexes. DHX9 contains double‐strand RNA bind domains 1 and 2 (dsRBD1, ds RBD2), a core helicase domain and C‐terminus of the repeated arginine and glycine‐glycine regions. The RGG region is necessary for nucleocytoplasmic shuttling in a RNA‐independent manner.^38^ Cristini A. et al identified that DHX9 was an important player in transcriptional termination and R‐loop‐associated DNA damage by RNA/DNA hybrid interactome, which could regulate R‐loop balance by interacting with PARP1.^16^ Our results demonstrated that C1orf109L could bind to the core helicase region and RGG region of DHX9. That is evidence that C1orf109L participates in the R‐loop regulatory complex and influences DHX9 function by interaction. Meanwhile, we also discovered that C1orf109L did not interact with PARP1 and SFPQ, which proteins bound to DHX9 and prevented R‐loop formation.^16^, ^18^ Our research data exhibited that C1orf109L bound to DHX9 by competitive with PARP1 and enhanced R‐loop accumulation, further indicated that C1orf109L could be impaired the role of regulating R‐loop formation by DHX9 binding with PARP1. Additionally, the data of PARP1 and DHX9 knocking down also verified that C1orf109L dependent on DHX9 to mediate DNA damage.

However, when SFPQ is knocked down to induce R‐loop formation first, DHX9 will promote R‐loop accumulation.^18^ It clarified that DHX9 function in regulating R‐loop could be modulated by multiple factors.

Enormous DNA damage was induced by R‐loop excessive accumulation, which may represent a novel way to promote cancer cell death in cancer therapy. CPT is a kind of anti‐tumour medicine and an R‐loop activator.^32^, ^39^ CPT can induce R‐loop accumulation, which promotes sustained γH2AX up‐regulation for 4 hours, followed by a rapid drop.^40^, ^41^ We found that C1orf109L could interact with DHX9 to promote R‐loop formation. The further results revealed that C1orf109L combined with CPT might lead to R‐loop accumulation and more serious DNA damage. It was reported that when CPT was used as a treatment agent, the deficiency of DHX9 could promote R‐loop accumulation and DNA damage.^16^ Actually, the expression of C1orf109L led to cell death in response to 5 hours of CPT treatment. And then, the cell death pathway was activated with CPT treatment in a time‐dependent manner. In the process, C1orf109L could enhance CPT chemosensitivity.

Therefore, C1orf109L interacting with DHX9 may regulate R‐loop formation, even could trigger R‐loop accumulation and further increase in response to CPT‐induced serious DNA damage. It will help us to understand the mechanism of regulation the R‐loop accumulation by C1orf109L, a candidate R‐loop‐associated protein.

## CONFLICT OF INTEREST

The authors declare that they have no competing interests.

## AUTHOR CONTRIBUTIONS

Yu LI conceived this study. Peng Dou and Yu LI designed the experiments. Peng Dou performed the main experiments. Haoxiu Sun finished the confirmation of protein interaction and immunofluorescence of siPARP1 and siDHX9. Yiqun Li and Xiaohan Zhang performed the mass spectrometry analyses and transcriptome analyses. Wanqiu Xie, Xiaoqing Zhang, Dandan Zhang, Shupei Qiao and Yanpeng Ci assisted in the cell cycle and immunofluorescence experiments. Fang Han and Huan Nie performed the data statistics and interpretation. Peng Dou, Fang Han and Yu Li wrote the manuscript. Huan Nie helped to revise the manuscript.

## Supporting information

Figure S1

Figure S2

Figure S3

Figure S4

Figure S5

Figure S6

Table S1‐S7

Data

Supplementary Material

## Data Availability

All data used during the study appear in the submitted article.
