# Supplementary figures and images for: C1orf109L binding DHX9 promotes DNA damage depended on the R‐loop accumulation and enhances camptothecin chemosensitivity
Source: Cell Prolif. 2020 Aug 6;53(9):e12875. doi: 10.1111/cpr.12875 (PMC7507383; doi:10.1111/cpr.12875)

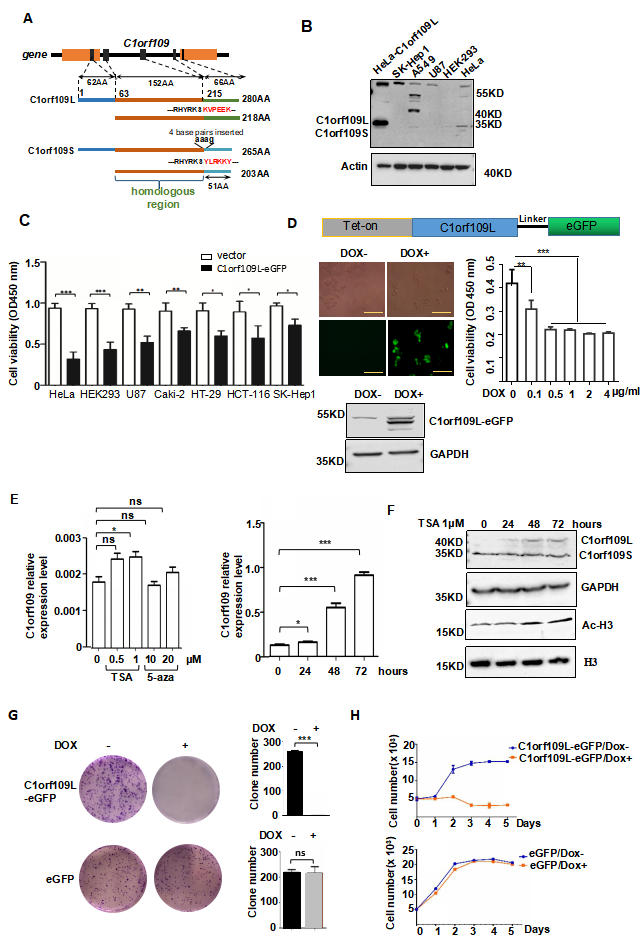

Supplement: Supplementary file 1 — Figure S1 [file CPR-53-e12875-s001.tif]

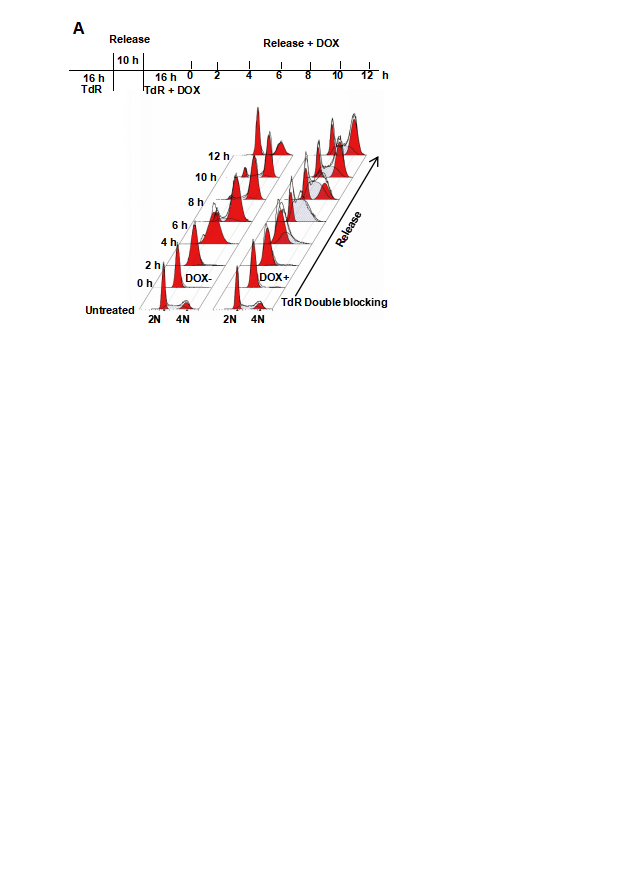

Supplement: Supplementary file 2 — Figure S2 [file CPR-53-e12875-s002.tif]

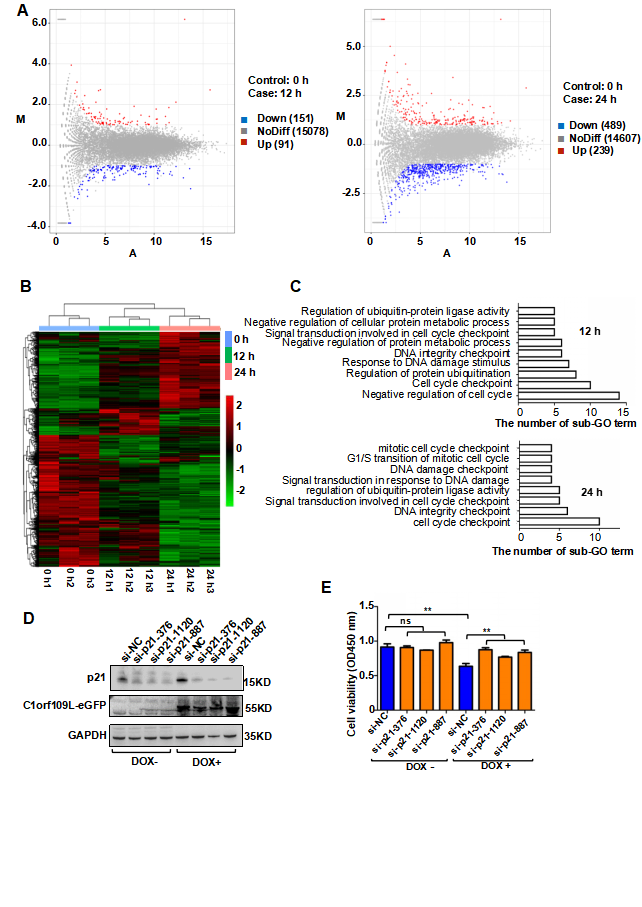

Supplement: Supplementary file 3 — Figure S3 [file CPR-53-e12875-s003.tif]

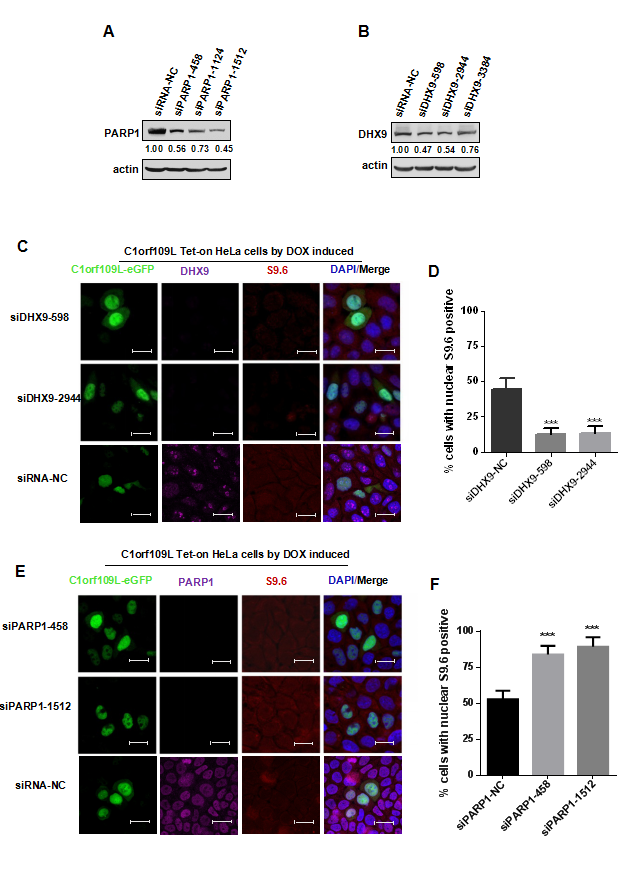

Supplement: Supplementary file 4 — Figure S4 [file CPR-53-e12875-s004.tif]

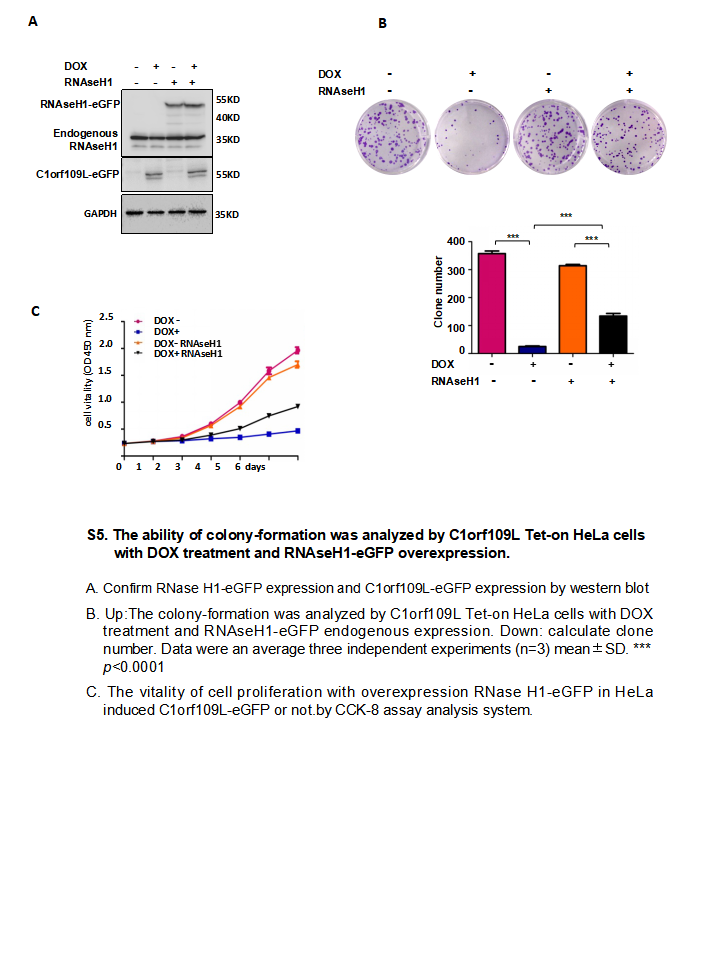

Supplement: Supplementary file 5 — Figure S5 [file CPR-53-e12875-s005.tif]

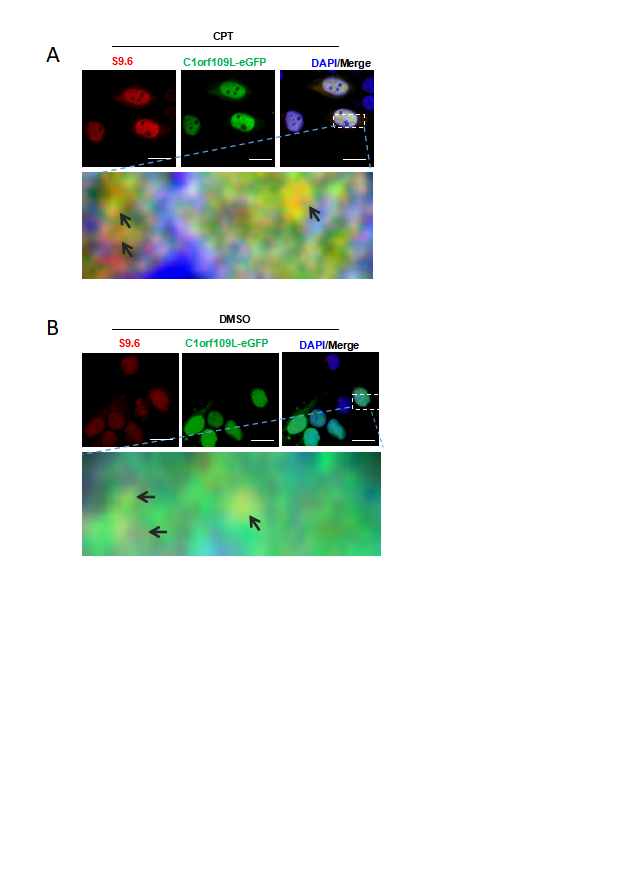

Supplement: Supplementary file 6 — Figure S6 [file CPR-53-e12875-s006.tif]
