# Supplementary material for: C1orf109L binding DHX9 promotes DNA damage depended on the R‐loop accumulation and enhances camptothecin chemosensitivity
Source: Cell Prolif. 2020 Aug 6;53(9):e12875. doi: 10.1111/cpr.12875 (PMC7507383; doi:10.1111/cpr.12875)
Supplement: Supplementary file 7 — Table S1‐S7 [file CPR-53-e12875-s007.docx]

**Table S1** Significant GO annotation results associated with cell cycle (12h)

| **GOID** | **Description** | ***p* value** | **q value** |
| --- | --- | --- | --- |
| GO:0072474 | signal transduction involved in mitotic cell cycle G1/S checkpoint | 6.85E-08 | 2.50E-05 |
| GO:0072395 | signal transduction involved in cell cycle checkpoint | 8.24E-08 | 2.50E-05 |
| GO:0072404 | signal transduction involved in G1/S transition checkpoint | 8.24E-08 | 2.50E-05 |
| GO:0000216 | M/G1 transition of mitotic cell cycle | 1.20E-07 | 2.80E-05 |
| GO:0031575 | mitotic cell cycle G1/S transition checkpoint | 1.20E-07 | 2.80E-05 |
| GO:0071779 | G1/S transition checkpoint | 1.40E-07 | 2.80E-05 |
| GO:2000045 | regulation of G1/S transition of mitotic cell cycle | 3.35E-07 | 4.82E-05 |
| GO:0071158 | positive regulation of cell cycle arrest | 3.73E-07 | 5.10E-05 |
| GO:0000082 | G1/S transition of mitotic cell cycle | 2.30E-06 | 0.000196 |
| GO:0051320 | S phase | 1.80E-05 | 0.001142 |
| GO:0007093 | mitotic cell cycle checkpoint | 2.79E-05 | 0.00173 |
| GO:0090068 | positive regulation of cell cycle process | 3.87E-05 | 0.0023 |
| GO:2000602 | regulation of interphase of mitotic cell cycle | 3.87E-05 | 0.0023 |
| GO:0000075 | cell cycle checkpoint | 8.05E-05 | 0.0044 |
| GO:0071156 | regulation of cell cycle arrest | 0.000169 | 0.009051 |
| GO:0045786 | negative regulation of cell cycle | 0.000356 | 0.01834 |
| GO:0007050 | cell cycle arrest | 0.000596 | 0.029097 |

**Table S2** Significant GO annotation results associated with cell cycle (24h)

| **GOID** | **Description** | ***p*value** | **q value** |
| --- | --- | --- | --- |
| GO:2000045 | regulation of G1/S transition of mitotic cell cycle | 1.87E-07 | 0.000243091 |
| GO:0031575 | mitotic cell cycle G1/S transition checkpoint | 5.58E-07 | 0.000258571 |
| GO:0072413 | signal transduction involved in mitotic cell cycle checkpoint | 6.59E-07 | 0.000258571 |
| GO:0072474 | signal transduction involved in mitotic cell cycle G1/S checkpoint | 6.59E-07 | 0.000258571 |
| GO:0071779 | G1/S transition checkpoint | 7.14E-07 | 0.000258571 |
| GO:0072395 | signal transduction involved in cell cycle checkpoint | 8.74E-07 | 0.000271264 |
| GO:0072404 | signal transduction involved in G1/S transition checkpoint | 8.74E-07 | 0.000271264 |
| GO:0000216 | M/G1 transition of mitotic cell cycle | 2.24E-06 | 0.000607614 |
| GO:0071158 | positive regulation of cell cycle arrest | 8.40E-06 | 0.001586983 |
| GO:0000082 | G1/S transition of mitotic cell cycle | 1.76E-05 | 0.003187774 |
| GO:2000602 | regulation of interphase of mitotic cell cycle | 2.71E-05 | 0.004201754 |
| GO:0007093 | mitotic cell cycle checkpoint | 0.000111 | 0.014131744 |
| GO:0000084 | S phase of mitotic cell cycle | 0.000188 | 0.020912944 |
| GO:0000075 | cell cycle checkpoint | 0.000454 | 0.048130253 |

**Table S3** Significant GO annotation results associated with DNA damage

| **GOID** | **Description** | ***p* value** | **q value** |
| --- | --- | --- | --- |
| GO:0031571 | mitotic cell cycle G1/S transition DNA damage checkpoint | 2.67E-08 | 2.50E-05 |
| GO:0006977 | DNA damage response, signal transduction by p53 class mediator resulting in cell cycle arrest | 6.85E-08 | 2.50E-05 |
| GO:0072401 | signal transduction involved in DNA integrity checkpoint | 6.85E-08 | 2.50E-05 |
| GO:0072422 | signal transduction involved in DNA damage checkpoint | 6.85E-08 | 2.50E-05 |
| GO:0072431 | signal transduction involved in mitotic cell cycle G1/S transition DNA damage checkpoint | 6.85E-08 | 2.50E-05 |
| GO:0000077 | DNA damage checkpoint | 6.66E-07 | 8.28E-05 |
| GO:0031570 | DNA integrity checkpoint | 1.37E-06 | 0.000138 |
| GO:0030330 | DNA damage response, signal transduction by p53 class mediator | 2.76E-06 | 0.000228 |
| GO:0042770 | signal transduction in response to DNA damage | 5.30E-06 | 0.000371 |
| GO:0006974 | response to DNA damage stimulus | 0.000542 | 0.027424 |

**Table S4** Significant GO annotation results associated with DNA damage

| **GOID** | **Description** | ***P* value** | **q value** |
| --- | --- | --- | --- |
| GO:0031571 | mitotic cell cycle G1/S transition DNA damage checkpoint | 2.24E-07 | 0.000243091 |
| GO:0006977 | DNA damage response, signal transduction by p53 class mediator resulting in cell cycle arrest | 6.59E-07 | 0.000258571 |
| GO:0072401 | signal transduction involved in DNA integrity checkpoint | 6.59E-07 | 0.000258571 |
| GO:0072422 | signal transduction involved in DNA damage checkpoint | 6.59E-07 | 0.000258571 |
| GO:0072431 | signal transduction involved in mitotic cell cycle G1/S transition DNA damage checkpoint | 6.59E-07 | 0.000258571 |
| GO:0042770 | signal transduction in response to DNA damage | 1.39E-06 | 0.000402136 |
| GO:0000077 | DNA damage checkpoint | 6.60E-06 | 0.001401298 |
| GO:0031570 | DNA integrity checkpoint | 2.01E-05 | 0.003491711 |
| GO:0030330 | DNA damage response, signal transduction by p53 class mediator | 2.26E-05 | 0.003772227 |

**Table S5** C1orf109L interaction with Proteins Summary

| **Number** | **Acc** | **#Peptide** | **#Unique Peptide** | **Coverage(%)** |
| --- | --- | --- | --- | --- |
| 1 | sp\|P16066\|ANPRA_HUMAN | 19 | 19 | 23.09 |
| 2 | sp\|Q92598\|HS105_HUMAN | 19 | 17 | 26.92 |
| 3 | sp\|O94826\|TOM70_HUMAN | 15 | 15 | 31.91 |
| 4 | sp\|Q9NX04\|CA109_HUMAN | 18 | 18 | 53.69 |
| 5 | sp\|Q71U36\|TBA1A_HUMAN | 13 | 4 | 36.36 |
| 6 | sp\|Q08211\|DHX9_HUMAN | 12 | 12 | 12.91 |
| 7 | sp\|O95757\|HS74L_HUMAN | 17 | 13 | 27.29 |
| 8 | sp\|Q9Y230\|RUVB2_HUMAN | 11 | 11 | 31.32 |
| 9 | sp\|Q9NVI7\|ATD3A_HUMAN | 10 | 4 | 21.14 |
| 10 | sp\|Q12797\|ASPH_HUMAN | 9 | 9 | 15.04 |
| 11 | sp\|P31948\|STIP1_HUMAN | 9 | 9 | 21.55 |
| 12 | sp\|Q96AG4\|LRC59_HUMAN | 9 | 9 | 32.25 |
| 13 | sp\|P26640\|SYVC_HUMAN | 7 | 7 | 7.75 |
| 14 | sp\|P25705\|ATPA_HUMAN | 7 | 7 | 15.37 |
| 15 | sp\|Q13724\|MOGS_HUMAN | 8 | 8 | 13.14 |
| 16 | sp\|P53621\|COPA_HUMAN | 7 | 7 | 6.94 |
| 17 | sp\|Q02878\|RL6_HUMAN | 7 | 7 | 31.94 |
| 18 | sp\|P46781\|RS9_HUMAN | 7 | 7 | 27.84 |
| 19 | sp\|P08240\|SRPRA_HUMAN | 7 | 7 | 15.99 |
| 20 | sp\|Q9Y4P3\|TBL2_HUMAN | 6 | 6 | 17.9 |
| 21 | sp\|P60709\|ACTB_HUMAN | 6 | 1 | 22.67 |
| 22 | sp\|Q15008\|PSMD6_HUMAN | 5 | 5 | 17.48 |
| 23 | sp\|Q16891\|MIC60_HUMAN | 6 | 6 | 10.42 |
| 24 | sp\|P04844\|RPN2_HUMAN | 5 | 5 | 13.31 |
| 25 | sp\|Q2TB90\|HKDC1_HUMAN | 5 | 5 | 7.74 |
| 26 | sp\|P16615\|AT2A2_HUMAN | 5 | 5 | 6.33 |
| 27 | sp\|P48651\|PTSS1_HUMAN | 5 | 5 | 10.78 |
| 28 | sp\|P23246\|SFPQ_HUMAN | 5 | 5 | 10.04 |
| 29 | sp\|Q14683\|SMC1A_HUMAN | 4 | 4 | 4.06 |
| 30 | sp\|P18621\|RL17_HUMAN | 5 | 5 | 23.37 |
| 31 | sp\|Q96TA2\|YMEL1_HUMAN | 4 | 4 | 6.99 |
| 32 | sp\|P50416\|CPT1A_HUMAN | 4 | 4 | 6.34 |
| 33 | sp\|P30050\|RL12_HUMAN | 4 | 4 | 33.94 |
| 34 | sp\|P62277\|RS13_HUMAN | 4 | 4 | 33.11 |
| 35 | sp\|P05023\|AT1A1_HUMAN | 4 | 4 | 5.47 |
| 36 | sp\|P62280\|RS11_HUMAN | 4 | 4 | 29.11 |
| 37 | sp\|O75439\|MPPB_HUMAN | 4 | 4 | 9 |
| 38 | sp\|Q12906\|ILF3_HUMAN | 5 | 5 | 8.5 |
| 39 | sp\|P16402\|H13_HUMAN | 4 | 4 | 12.67 |
| 40 | sp\|P62244\|RS15A_HUMAN | 4 | 4 | 32.31 |
| 41 | sp\|P52292\|IMA1_HUMAN | 4 | 4 | 12.85 |
| 42 | sp\|Q99459\|CDC5L_HUMAN | 3 | 3 | 4.36 |
| 43 | sp\|P40227\|TCPZ_HUMAN | 4 | 4 | 11.3 |
| 44 | sp\|Q9NXS2\|QPCTL_HUMAN | 3 | 3 | 8.12 |
| 45 | sp\|Q9UJS0\|CMC2_HUMAN | 3 | 3 | 6.37 |
| 46 | sp\|Q13045\|FLII_HUMAN | 3 | 3 | 2.84 |
| 47 | sp\|P53618\|COPB_HUMAN | 3 | 3 | 4.41 |
| 48 | sp\|Q08379\|GOGA2_HUMAN | 3 | 3 | 3.49 |
| 49 | sp\|P40429\|RL13A_HUMAN | 3 | 3 | 14.29 |
| 50 | sp\|P35998\|PRS7_HUMAN | 3 | 3 | 9.01 |
| 51 | sp\|P39019\|RS19_HUMAN | 3 | 3 | 24.83 |
| 52 | sp\|P30453\|1A34_HUMAN | 3 | 3 | 11.51 |
| 53 | sp\|P62979\|RS27A_HUMAN | 3 | 3 | 30.77 |
| 54 | sp\|P52597\|HNRPF_HUMAN | 5 | 3 | 17.59 |
| 55 | sp\|Q9UQE7\|SMC3_HUMAN | 3 | 3 | 3.04 |
| 56 | sp\|P53007\|TXTP_HUMAN | 3 | 3 | 10.29 |
| 57 | sp\|Q9NRJ1\|MOST1_HUMAN | 3 | 3 | 11.11 |
| 58 | sp\|P62888\|RL30_HUMAN | 3 | 3 | 34.78 |
| 59 | sp\|Q01844\|EWS_HUMAN | 3 | 3 | 7.32 |
| 60 | sp\|O43390\|HNRPR_HUMAN | 9 | 4 | 15.01 |
| 61 | sp\|Q8TCJ2\|STT3B_HUMAN | 3 | 3 | 4.12 |
| 62 | sp\|Q86UE4\|LYRIC_HUMAN | 3 | 3 | 8.08 |
| 63 | sp\|P55209\|NP1L1_HUMAN | 3 | 3 | 9.72 |
| 64 | sp\|P06748\|NPM_HUMAN | 3 | 3 | 24.49 |
| 65 | sp\|Q9UNX3\|RL26L_HUMAN | 3 | 3 | 17.24 |
| 66 | sp\|P63244\|RACK1_HUMAN | 4 | 4 | 16.72 |
| 67 | sp\|Q9UKS6\|PACN3_HUMAN | 3 | 3 | 9.67 |
| 68 | sp\|Q9NR30\|DDX21_HUMAN | 2 | 2 | 3.45 |
| 69 | sp\|Q9NUL7\|DDX28_HUMAN | 3 | 3 | 6.67 |
| 70 | sp\|Q96A33\|CCD47_HUMAN | 3 | 3 | 7.45 |
| 71 | sp\|P26373\|RL13_HUMAN | 3 | 3 | 14.22 |
| 72 | sp\|O95573\|ACSL3_HUMAN | 2 | 2 | 4.17 |
| 73 | sp\|Q9UDY2\|ZO2_HUMAN | 3 | 3 | 3.53 |
| 74 | sp\|Q9Y5B9\|SP16H_HUMAN | 4 | 4 | 4.87 |
| 75 | sp\|Q92945\|FUBP2_HUMAN | 3 | 3 | 5.49 |
| 76 | sp\|P51648\|AL3A2_HUMAN | 3 | 3 | 8.04 |
| 77 | sp\|Q70UQ0\|IKIP_HUMAN | 2 | 2 | 6 |
| 78 | sp\|P62263\|RS14_HUMAN | 3 | 3 | 24.5 |
| 79 | sp\|Q9Y3U8\|RL36_HUMAN | 2 | 2 | 21.9 |
| 80 | sp\|Q9NZB2\|F120A_HUMAN | 2 | 2 | 2.42 |
| 81 | sp\|P27635\|RL10_HUMAN | 2 | 2 | 6.54 |
| 82 | sp\|Q9NVN8\|GNL3L_HUMAN | 2 | 2 | 5.67 |
| 83 | sp\|P50914\|RL14_HUMAN | 3 | 3 | 16.28 |
| 84 | sp\|P51398\|RT29_HUMAN | 2 | 2 | 7.29 |
| 85 | sp\|Q13310\|PABP4_HUMAN | 10 | 3 | 17.86 |
| 86 | sp\|P61313\|RL15_HUMAN | 2 | 2 | 10.29 |
| 87 | sp\|P62917\|RL8_HUMAN | 2 | 2 | 11.28 |
| 88 | sp\|P42766\|RL35_HUMAN | 2 | 2 | 21.95 |
| 89 | sp\|P84098\|RL19_HUMAN | 2 | 2 | 13.27 |
| 90 | sp\|Q9Y2R9\|RT07_HUMAN | 2 | 2 | 10.33 |
| 91 | sp\|Q96SB4\|SRPK1_HUMAN | 2 | 2 | 3.66 |
| 92 | sp\|P61353\|RL27_HUMAN | 2 | 2 | 14.71 |
| 93 | sp\|P08195\|4F2_HUMAN | 2 | 2 | 4.13 |
| 94 | sp\|Q9Y6H1\|CHCH2_HUMAN | 2 | 2 | 27.15 |
| 95 | sp\|Q5F1R6\|DJC21_HUMAN | 2 | 2 | 5.84 |
| 96 | sp\|Q01804\|OTUD4_HUMAN | 2 | 2 | 3.95 |
| 97 | sp\|P46778\|RL21_HUMAN | 2 | 2 | 20.62 |
| 98 | sp\|Q9P035\|HACD3_HUMAN | 2 | 2 | 8.01 |
| 99 | sp\|P30101\|PDIA3_HUMAN | 2 | 2 | 4.95 |
| 100 | sp\|Q8WWY3\|PRP31_HUMAN | 2 | 2 | 5.61 |
| 101 | sp\|Q8NBX0\|SCPDL_HUMAN | 2 | 2 | 6.29 |
| 102 | sp\|Q9Y2G8\|DJC16_HUMAN | 2 | 2 | 4.48 |
| 103 | sp\|P26196\|DDX6_HUMAN | 2 | 2 | 5.38 |
| 104 | sp\|Q9UNM6\|PSD13_HUMAN | 2 | 2 | 9.57 |
| 105 | sp\|O15397\|IPO8_HUMAN | 2 | 2 | 2.12 |
| 106 | sp\|Q92552\|RT27_HUMAN | 2 | 2 | 5.56 |
| 107 | sp\|P62191\|PRS4_HUMAN | 2 | 2 | 7.73 |
| 108 | sp\|P54652\|HSP72_HUMAN | 20 | 3 | 24.57 |
| 109 | sp\|O95470\|SGPL1_HUMAN | 2 | 2 | 5.63 |
| 110 | sp\|P27824\|CALX_HUMAN | 2 | 2 | 3.88 |
| 111 | sp\|O75533\|SF3B1_HUMAN | 2 | 2 | 2.45 |
| 112 | sp\|P50579\|MAP2_HUMAN | 2 | 2 | 5.65 |
| 113 | sp\|P28288\|ABCD3_HUMAN | 2 | 2 | 4.25 |
| 114 | sp\|Q9NYK5\|RM39_HUMAN | 2 | 2 | 7.1 |
| 115 | sp\|Q96PK6\|RBM14_HUMAN | 2 | 2 | 3.59 |
| 116 | sp\|Q2NL82\|TSR1_HUMAN | 3 | 3 | 4.98 |
| 117 | sp\|Q5T9A4\|ATD3B_HUMAN | 8 | 2 | 16.98 |
| 118 | sp\|Q9Y4W6\|AFG32_HUMAN | 2 | 2 | 3.76 |
| 119 | sp\|Q9UNE7\|CHIP_HUMAN | 2 | 2 | 8.91 |
| 120 | sp\|P52272\|HNRPM_HUMAN | 1 | 1 | 1.51 |
| 121 | sp\|P46087\|NOP2_HUMAN | 2 | 2 | 3.57 |
| 122 | sp\|O43809\|CPSF5_HUMAN | 2 | 2 | 11.89 |
| 123 | sp\|P11586\|C1TC_HUMAN | 3 | 3 | 4.28 |
| 124 | sp\|P14868\|SYDC_HUMAN | 2 | 2 | 3.79 |
| 125 | sp\|P50402\|EMD_HUMAN | 2 | 2 | 11.42 |
| 126 | sp\|P35613\|BASI_HUMAN | 1 | 1 | 3.64 |
| 127 | sp\|P08621\|RU17_HUMAN | 1 | 1 | 2.52 |
| 128 | sp\|Q16576\|RBBP7_HUMAN | 1 | 1 | 3.06 |
| 129 | sp\|O94874\|UFL1_HUMAN | 1 | 1 | 2.02 |
| 130 | sp\|P46776\|RL27A_HUMAN | 1 | 1 | 7.43 |
| 131 | sp\|Q96EY1\|DNJA3_HUMAN | 2 | 2 | 5.83 |
| 132 | sp\|Q96HS1\|PGAM5_HUMAN | 1 | 1 | 2.08 |
| 133 | sp\|Q14697\|GANAB_HUMAN | 1 | 1 | 1.8 |
| 134 | sp\|Q99832\|TCPH_HUMAN | 1 | 1 | 2.21 |
| 135 | sp\|Q6PKG0\|LARP1_HUMAN | 2 | 2 | 1.82 |
| 136 | sp\|Q00341\|VIGLN_HUMAN | 1 | 1 | 0.79 |
| 137 | sp\|Q07955\|SRSF1_HUMAN | 1 | 1 | 4.03 |
| 138 | sp\|Q86UP2\|KTN1_HUMAN | 1 | 1 | 0.96 |
| 139 | sp\|P62195\|PRS8_HUMAN | 1 | 1 | 3.45 |
| 140 | sp\|P63010\|AP2B1_HUMAN | 1 | 1 | 1.28 |
| 141 | sp\|P11387\|TOP1_HUMAN | 1 | 1 | 2.61 |
| 142 | sp\|Q9H078\|CLPB_HUMAN | 1 | 1 | 1.7 |
| 143 | sp\|Q9NY93\|DDX56_HUMAN | 1 | 1 | 2.56 |
| 144 | sp\|P14625\|ENPL_HUMAN | 3 | 1 | 3.49 |
| 145 | sp\|Q96HY6\|DDRGK_HUMAN | 1 | 1 | 4.46 |
| 146 | sp\|P68366\|TBA4A_HUMAN | 11 | 2 | 29.91 |
| 147 | sp\|P51991\|ROA3_HUMAN | 1 | 1 | 5.82 |
| 148 | sp\|Q9UG63\|ABCF2_HUMAN | 1 | 1 | 1.77 |
| 149 | sp\|Q9H6T3\|RPAP3_HUMAN | 1 | 1 | 2.26 |
| 150 | sp\|Q13895\|BYST_HUMAN | 1 | 1 | 4.58 |
| 151 | sp\|Q9Y3F4\|STRAP_HUMAN | 1 | 1 | 4.29 |
| 152 | sp\|Q9BZF1\|OSBL8_HUMAN | 1 | 1 | 1.24 |
| 153 | sp\|P55084\|ECHB_HUMAN | 2 | 2 | 3.8 |
| 154 | sp\|P60900\|PSA6_HUMAN | 1 | 1 | 5.29 |
| 155 | sp\|P35268\|RL22_HUMAN | 1 | 1 | 8.59 |
| 156 | sp\|Q14444\|CAPR1_HUMAN | 2 | 2 | 7.47 |
| 157 | sp\|P55060\|XPO2_HUMAN | 1 | 1 | 1.75 |
| 158 | sp\|Q15393\|SF3B3_HUMAN | 2 | 2 | 2.47 |
| 159 | sp\|P17342\|ANPRC_HUMAN | 1 | 1 | 3.88 |
| 160 | sp\|Q9P2J5\|SYLC_HUMAN | 1 | 1 | 1.02 |
| 161 | sp\|O95816\|BAG2_HUMAN | 2 | 2 | 15.64 |
| 162 | sp\|P60866\|RS20_HUMAN | 1 | 1 | 12.61 |
| 163 | sp\|Q9H0D6\|XRN2_HUMAN | 1 | 1 | 1.26 |
| 164 | sp\|Q32MZ4\|LRRF1_HUMAN | 1 | 1 | 1.98 |
| 165 | sp\|A2A3N6\|PIPSL_HUMAN | 1 | 1 | 1.62 |
| 166 | sp\|P43686\|PRS6B_HUMAN | 1 | 1 | 4.54 |
| 167 | sp\|P41252\|SYIC_HUMAN | 1 | 1 | 0.95 |
| 168 | sp\|Q15392\|DHC24_HUMAN | 1 | 1 | 1.74 |
| 169 | sp\|P05386\|RLA1_HUMAN | 1 | 1 | 14.04 |
| 170 | sp\|P00533\|EGFR_HUMAN | 1 | 1 | 1.57 |
| 171 | sp\|Q86UK7\|ZN598_HUMAN | 1 | 1 | 1.55 |
| 172 | sp\|Q8IWX8\|CHERP_HUMAN | 1 | 1 | 1.2 |
| 173 | sp\|P53985\|MOT1_HUMAN | 1 | 1 | 2.4 |
| 174 | sp\|Q71UM5\|RS27L_HUMAN | 1 | 1 | 15.48 |
| 175 | sp\|P23284\|PPIB_HUMAN | 1 | 1 | 6.02 |
| 176 | sp\|P62857\|RS28_HUMAN | 1 | 1 | 17.39 |
| 177 | sp\|P25398\|RS12_HUMAN | 1 | 1 | 6.82 |
| 178 | sp\|Q92896\|GSLG1_HUMAN | 1 | 1 | 1.27 |
| 179 | sp\|Q52LJ0\|FA98B_HUMAN | 1 | 1 | 3.64 |
| 180 | sp\|Q9H074\|PAIP1_HUMAN | 1 | 1 | 2.5 |
| 181 | sp\|Q14681\|KCTD2_HUMAN | 1 | 1 | 8.37 |
| 182 | sp\|Q9P0L0\|VAPA_HUMAN | 1 | 1 | 5.62 |
| 183 | sp\|P36542\|ATPG_HUMAN | 1 | 1 | 4.03 |
| 184 | sp\|Q04837\|SSBP_HUMAN | 1 | 1 | 10.14 |
| 185 | sp\|Q6P087\|RUSD3_HUMAN | 1 | 1 | 4.27 |
| 186 | sp\|P29317\|EPHA2_HUMAN | 1 | 1 | 1.64 |
| 187 | sp\|P29692\|EF1D_HUMAN | 1 | 1 | 4.27 |
| 188 | sp\|Q8WU90\|ZC3HF_HUMAN | 1 | 1 | 3.29 |
| 189 | sp\|Q5SRD1\|TI23B_HUMAN | 1 | 1 | 6.62 |
| 190 | sp\|P05109\|S10A8_HUMAN | 1 | 1 | 11.83 |
| 191 | sp\|O43684\|BUB3_HUMAN | 1 | 1 | 4.27 |
| 192 | sp\|Q8NFW8\|NEUA_HUMAN | 1 | 1 | 3.69 |
| 193 | sp\|Q15165\|PON2_HUMAN | 1 | 1 | 4.52 |
| 194 | sp\|Q15233\|NONO_HUMAN | 1 | 1 | 4.67 |
| 195 | sp\|P51665\|PSMD7_HUMAN | 1 | 1 | 4.01 |
| 196 | sp\|P10644\|KAP0_HUMAN | 1 | 1 | 4.46 |
| 197 | sp\|P78362\|SRPK2_HUMAN | 1 | 1 | 1.74 |
| 198 | sp\|Q07021\|C1QBP_HUMAN | 1 | 1 | 7.09 |
| 199 | sp\|Q9Y5Q9\|TF3C3_HUMAN | 1 | 1 | 1.69 |
| 200 | sp\|Q29RF7\|PDS5A_HUMAN | 1 | 1 | 0.97 |
| 201 | sp\|Q9UNF1\|MAGD2_HUMAN | 1 | 1 | 5.61 |
| 202 | sp\|P12236\|ADT3_HUMAN | 5 | 2 | 18.46 |
| 203 | sp\|Q92973\|TNPO1_HUMAN | 1 | 1 | 1.67 |
| 204 | sp\|P08708\|RS17_HUMAN | 1 | 1 | 8.89 |
| 205 | sp\|P81605\|DCD_HUMAN | 1 | 1 | 12.73 |
| 206 | sp\|P55036\|PSMD4_HUMAN | 1 | 1 | 2.65 |
| 207 | sp\|Q15629\|TRAM1_HUMAN | 1 | 1 | 3.48 |
| 208 | sp\|P25786\|PSA1_HUMAN | 1 | 1 | 5.7 |
| 209 | sp\|Q9BSJ8\|ESYT1_HUMAN | 1 | 1 | 1.18 |
| 210 | sp\|P21333\|FLNA_HUMAN | 1 | 1 | 0.64 |
| 211 | sp\|Q9UM00\|TMCO1_HUMAN | 1 | 1 | 7.98 |
| 212 | sp\|P62136\|PP1A_HUMAN | 1 | 1 | 5.45 |
| 213 | sp\|P43307\|SSRA_HUMAN | 1 | 1 | 3.85 |
| 214 | sp\|O95347\|SMC2_HUMAN | 1 | 1 | 1.09 |
| 215 | sp\|P39656\|OST48_HUMAN | 1 | 1 | 2.41 |
| 216 | sp\|Q9Y5M8\|SRPRB_HUMAN | 2 | 2 | 11.44 |
| 217 | sp\|O95793\|STAU1_HUMAN | 2 | 2 | 5.03 |
| 218 | sp\|P07237\|PDIA1_HUMAN | 1 | 1 | 2.16 |
| 219 | sp\|Q9Y2R4\|DDX52_HUMAN | 1 | 1 | 3.01 |
| 220 | sp\|Q92900\|RENT1_HUMAN | 1 | 1 | 0.97 |
| 221 | sp\|Q14157\|UBP2L_HUMAN | 1 | 1 | 1.66 |
| 222 | sp\|P83111\|LACTB_HUMAN | 1 | 1 | 2.19 |
| 223 | sp\|P35232\|PHB_HUMAN | 1 | 1 | 3.68 |
| 224 | sp\|Q9Y6K5\|OAS3_HUMAN | 2 | 2 | 1.66 |
| 225 | sp\|P31689\|DNJA1_HUMAN | 1 | 1 | 3.27 |
| 226 | sp\|O00411\|RPOM_HUMAN | 1 | 1 | 0.89 |
| 227 | sp\|P60891\|PRPS1_HUMAN | 1 | 1 | 3.77 |
| 228 | sp\|Q14318\|FKBP8_HUMAN | 1 | 1 | 3.4 |
| 229 | sp\|Q13247\|SRSF6_HUMAN | 1 | 1 | 2.62 |
| 230 | sp\|Q9BUF5\|TBB6_HUMAN | 6 | 1 | 13.68 |
| 231 | sp\|Q6P4Q7\|CNNM4_HUMAN | 1 | 1 | 1.81 |
| 232 | sp\|P78347\|GTF2I_HUMAN | 1 | 1 | 1.7 |
| 233 | sp\|Q9BXS6\|NUSAP_HUMAN | 1 | 1 | 4.54 |
| 234 | sp\|Q99700\|ATX2_HUMAN | 1 | 1 | 1.14 |
| 235 | sp\|Q96JB5\|CK5P3_HUMAN | 1 | 1 | 2.77 |
| 236 | sp\|P15311\|EZRI_HUMAN | 1 | 1 | 2.56 |

| **Table S6**. The interaction protein known to bind an RNA/DNA hybrid in the C1orf109L interaction proteome | | | | | |
| --- | --- | --- | --- | --- | --- |
|  | **Protein** | **C1orf109L IP-MS** | | **R-loop interactome from PDX002960^[16]^** | |
|  |  | **Unused** | **Unique Peptide** | **Corrected p-value** | **Unique Peptide** |
| 1 | DHX9 | 24.58 | 12 | 1.11E-06 | 45.2 |
| 2 | SFPQ | 8.57 | 5 | 5.80E-05 | 13.4 |
| 3 | CDC5L | 6.61 | 3 | 1.26E-05 | 20.7 |
| 4 | HNRNPR(HNRPR) | 5.92 | 4 | 1.06E-06 | 32.2 |
| 5 | NPM1(NPM) | 5.40 | 3 | 1.08E-04 | 38.8 |
| 6 | DDX6 | 4.00 | 2 | 9.45E-05 | 5.2 |
| 7 | SRPK1 | 4.00 | 2 | 1.10E-05 | 12.7 |
| 8 | RBM14 | 3.43 | 2 | 1.82E-05 | 7.8 |
| 9 | HNRNPM(HNRPM) | 3.09 | 1 | 1.87E-06 | 37.5 |
| 10 | NOP2 | 3.02 | 2 | 7.91E-07 | 45.2 |
| 11 | SRSF1 | 2.13 | 1 | 7.91E-07 | 46.4 |
| 12 | TOP1 | 2.11 | 1 | 1.83E-04 | 41 |
| 13 | DDX56 | 2.10 | 1 | 1.87E-06 | 39.1 |
| 14 | XRN2 | 2.01 | 1 | 4.10E-06 | 30.9 |
| 15 | NONO | 2.00 | 1 | 4.33E-06 | 36.3 |
| 16 | SRPK2 | 2.00 | 1 | 2.58E-04 | 8.1 |
| 17 | DDX52 | 1.77 | 1 | 7.17E-05 | 5.5 |
| 18 | UPF1(RENT1) | 1.76 | 1 | 2.88E-06 | 29.2 |
| 19 | SRSF6 | 1.51 | 1 | 1.42E-04 | 25 |

Table S7 siRNAs and primers sequence

| siRNA | 5′-3′ sequence |  |
| --- | --- | --- |
| NC for sip21 | UUCUUCCGAACGUGUCACGUTT |  |
| sip21-376 | GAUGGAACUUCGACUUUGUTT |  |
| sip21-887 | CCUCUGGCAUUAGAAUUAUTT |  |
| sip21-1120 | CAGGCGGUUAUUGAAAUUCATT |  |
|  |  |  |
| NC for siPARP1 | UUCUUCCGAACGUGUCACGUTT |  |
| siPARP1-458 | GCAAAGGCCAGGAUGGAAUTT |  |
| siPARP1-1124 | GGACCAAGUGUAUGGUCAATT |  |
| siPARP1-1512 | GGAAGCCAACAUCCGAGUUTT |  |
|  |  |  |
| NC for siDHX9 | UUCUUCCGAACGUGUCACGUTT |  |
| siDHX9-2944 | CCUGGGAUGAUGCUAGAAUTT |  |
| siDHX9-598 | GAGCCAACUUGAAGGAUUATT |  |
| siDHX9-3384 | GCCUCCAAGAAAGUCCAAUTT |  |
|  |  |  |
| RT-PCR primer |  |  |
| C1orf109 |  |  |
| sense | CTGCGGTTTGAGGATGTGC |  |
| anti-sense | CTAGCTTGTCCAGGACGATGT |  |
|  |  |  |
| Tet-on C1orf109L construct primer | |  |
| sense | CGACCGGTGCCACCATGTCAGAGAAGAGACGGCGC | |
| anti-sense | CCATCGATCTACAGCTCGTCCATGCCGA |  |
|  |  |  |
| RnaseH1 construct primer | |  |
| sense | CGGAATTCATGTTCTATGCCGTGAGGAGG |  |
| anti-sense | GCCTCGAGGTCTTCCGATTGTTTAGCTCCTT | |
