# Supplementary material for: C1orf109L binding DHX9 promotes DNA damage depended on the R‐loop accumulation and enhances camptothecin chemosensitivity
Source: Cell Prolif. 2020 Aug 6;53(9):e12875. doi: 10.1111/cpr.12875 (PMC7507383; doi:10.1111/cpr.12875)
Supplement: Supplementary file 9 — Supplementary Material [file CPR-53-e12875-s009.docx]

**reagents**

For antibody, mouse anti-Clorf109 monoclonal antibody was purchased from Genscript Company (Nanjing, China. WB 1:1000 dilution), rabbit anti-eGFP polyclonal antibody (Abcam, WB 1:1000 dilution and IP: 2 μg/ml, ab290), rabbit anti-PARP1 monoclonal antibody (Cell Signaling technology, WB 1:1000, Lot#9532), rabbit anti-phospho-Histone H2A.X (Ser139, 20E3) monoclonal antibody (Cell Signaling technology, WB 1:1000 dilution, Lot#9718), mouse anti-DHX9 polyclonal antibody (Abcam, WB 1:1000 dilution, ab26271), mouse anti-p21 monoclonal antibody (Proteintech, WB 1:1000, Catalog No: 60214-1-Ig). mouse anti-phospho-CDK1-T14 monoclonal antibody (ABclonal, WB 1:1000 dilution, Catalog No: AP0015), rabbit anti-RNAseH1 polyclonal antibody (ABclonal, WB 1:1000 dilution, Catalog No: A9116), anti-GAPDH (ABclonal, WB 1:50000 dilution, AC033), mouse anti-S9.6 monoclonal antibody (EMD millipore, IF 1:50 dilution, Lot#301193 ), mouse Flag-Tag monoclonal antibody (Thermo Fisher, WB 1:1000 dilution, Catalog No: MA1-91878), rabbit anti-Histone H3 polyclonal antibody (Proteintech, WB 1:1000, Catalog No: 17168-1-AP), rabbit anti-NPM1 (N-term) polyclonal antibody (Absin, WB 1:1000, abs106184).

For reagents, Campathecin (MCE, Catalog No: HY-16560), Nocodazle (MCE, Catalog No: HY-13520), Doxycycline (MCE, Catalog No: HY-N05658), Phosphatase inhibitor cocktail 1 (MCE, Catalog No: HY-K0022), protease inhibitor cocktail 1 (MCE, Catalog No: HY-K0010) protein A/G magnetic beads (MCE, HY-K0202). Flag-M2 magnetic beads (Theromo fisher, Lot: TJ276517)

**Plasmid constructs and siRNA**

The recombinants of pTRIPZ-C1orf109L-eGFP, pLVSIN-RNaseH1-eGFP and pCMV-Flag-C1orf109L were constructed. Briefly, pLVSIN plasmid and pCMV-Flag were digested with EcoR I/Xho I at 37℃ for 1 hour, the same condition was applied to digest pTRIPZ with Age I/Cla I, then desired digests were isolated by 1% agarose gel electrophoresis. The bands were excised and purified with Gel Extraction Kit (Theromo Fisher). For inserts, we get inserts from cDNA of HEK-293 by PCR. According to the need to construct the vector, inserts digest with the corresponding restriction endonuclease at 37℃ for 1 hour, the desired inserts were purified with PCR Purification Kit (Theromo Fisher). The ligation reactions of insert with 50 ng vector at a 3:1 molar ratio were performed at room temperature for 1 hour using T4-DNA ligase and buffer in a final volume of 10 μL. Ligation mixtures were used to transform the DH5α following the manufacturer’s instructions. 50 μL of transformation mixture were plated onto LB-agar containing ampicillin. 12 hours later, the [select](../../AppData/Local/youdao/dict/Application/7.5.2.0/resultui/dict/%3fkeyword=select)ed clones and [amplifie](../../AppData/Local/youdao/dict/Application/7.5.2.0/resultui/dict/%3fkeyword=amplification)d with LB containing ampicillin. Finally, extract the plasmid from bacteria solution.

Ablation of p21, DHX9 and PARP1 was performed by transfection with siRNA duplex oligos, which were synthesized by GenePharma Company (Shanghai, China). The sequences of the siRNAs and related primer were as [Table](../../AppData/Local/youdao/dict/Application/7.5.2.0/resultui/dict/%3fkeyword=table) S7. Cell transfection was performed with Lipofectamine TM 2000 (Invitrogen) as described in the manufacturer’s protocol.

**Lentiviral Production**

Human C1orf109L-eGFP Lentiviral Vector was constructed (pTRIPZ-C1orf109L

-eGFP) and was used to overexpress human C1orf109L in HeLa and HEK-293. Viral particles were produced with a HEK-293T packaging cell lines, cells were infected once with viral supernatants. At day 2, infected cells were selected with puromycin for 7 days and placed in experiments. Overexpression of C1orf109L-eGFP was checked by Western blot and fluorescence inverted microscope (Olympus IX71).

**Silver staining**

After finished SDS-PAGE, we used Protein Silver Stain Kit stained the gel, Briefly, The procedure consisted soffixing with methanol, acetic acid and paraformaldehyde solutions, washed with ethanol (50% and 30%) and ddH_2_O, sensitizing with Na_2_S_2_O_3_.5H_2_O, washed with ddH_2_O, impregnating with silver nitrate and paraformaldehyde solution, washed with ddH_2_O, developing with Na_2_CO_3_, paraformalde-hyde and Na_2_S_2_O_3_.5H_2_O solution, washed with ddH_2_O, and ending reaction with a stop solution-methanol 50%, and acetic acid 12%. Images were acquired with a camera (Cannon)

**PCR experiments**

Total RNA was purified. Briefly, nucleic acids were extracted using Trizol reagent, Then, 1μg of total RNA was subjected to reverse transcription with random primers using Takera’s TranscriptorFirst Strand cDNA Synthesis Kit and cDNA was subjected to PCR analyses.

**Cell proliferation assays**

Tet-on HeLa and Tet-on HEK-293 cells were seeded into 96-well plates with a density of 5,000 cells per well overnight (time 0) and treated with DOX (500 ng/mL) or DMSO. After indicated time treatment, a mixed solution consisting of CCK-8 (10 µL, MCE) and fresh culture medium (100 µL) was added to each well and incubated for an additional 2 hours at 37℃ and 5% CO_2_. Finally, the absorbance at 450 nm was measured by a microplate reader (BioTek Synergy TM2). For colony formation assays, cells were seeded in six-well plate sat a density of 1000 cells per well treated with DOX or DMSO and cultured at 37℃ for two weeks. After incubation, the cells were fixed with 100% methanol and stained with 0.1% (w/v) Crystal Violet. Pictures were captured with camera (Cannon), and the zoom picture were acquired with stereoscopic microscope (Olympus SZX10)

**cell cycle synchronization**

For cell cycle synchronization, overnight post-plating, 2 mM Thymidine (TdR) was added to the culture medium. Following 16 hours incubation, cells were washed and fresh medium was added. Following 10 hours incubation, 2 mM Thymidine was again added to the culture medium and the cells incubated for a further 16 hours. Cells were then washed and contain DOX or DMSO media added. Following release from Thymidine block, for every indicated time, collect cell to detect cell cycle.

**Immunofluorescence**

Tet-on HeLa cells were seeded with a density of 1×10^5^ per well in 12-well plates. After incubation for 24 hours, the cells were treated with DOX or DMSO and further incubated for 24 hours at 37℃ and 5% CO_2_. Then, the cells on the slides were fixed with 4% PFA at room temperature for 10 minutes and permeabilized with 0.2% Triton X-100 at 37℃ for 10 minutes, followed by incubation with anti-S9.6 antibody (dilution at 1:50) at room temperature for 60 minutes. Finally, the preparations were washed with PBS and mounted in fluorescent mounting medium with DAPI (Invitrogen). Negative controls were processed in the same way but without the primary antibody. Slides were photographed under a laser scanning confocal microscopy (Zeiss LSM510).

**Live cell imaging**

Cells were plated on 35cm glass bottom dishes (MatTek). Imaging experiments were performed from 0 to 8 hours, for every 6 minutes collect a picture, with or without DOX induction for 24 hours. For CPT treatment experiment, Tet-on HeLa C1orf109L-eGFP and Tet-on HeLa RFP cells were plated on 35 cm glass bottom dishes with same number overnight. Treated cells with DOX and perform live cell imaging. 488 nm (identify C1orf109L-eGFP expression cells) and 563 nm (identify RFP expression cells) channel were used. Imaging experiments were performed from 0 to 8 hours, every 6 minutes picture was captured. Image acquisition was performed using a live cell Imaging System (Observer Z1).

**Creating and analyzing protein-protein interaction networks**

Protein-protein interactions (PPI) among identified C1orf109L binding partners were extracted using the Search Tool for the Retrieval of Interacting Genes/Proteins (RRID: SCR_005223). The protein-protein interactions (PPI) were constructed by STRING website, only PPIs (*p*<0.001) from curated databases or curated published experiments were included in the PPI retrieval, and a minimum integrated confidence score of 0.5 was required for each interaction. Identified interactions were visualized using Cytoscape. We used the R-package “cluster-profiler” to perform GO annotation enrichment analysis, the *p* value was justified by the “Benjamini & Hochberg, BH” method.^42^ The PPI network edge thickness represents the integrated confidence score for the interaction (ranging for medium confidence score of 0.5 to high confidence score of 1). Node colour in gray scale from light to dark indicates increasing abundance of the interactor in the C1of109L-eGFP immunoprecipitation, based on coverage rate of unique peptides (unique peptides number/length of protein) in LC-MS/MS data (ranging from 0.0007 to 0.006). Network and node level statistics were extracted from the resulting network using Cytoscape.

**Comet assay**

After treatment with or without DOX for 24 hours, cells were collected for analyzing DNA damage activity by comet assay. Cell density was adjusted to 1000 cells mixed with 0.5% low-melting point agarose equilibrated to 37℃, and cell-agarose suspensions were spread onto the comet slides embedded with 1% normal-melting agarose for incubation at 4℃. The prepared slides were lysed immediately in chilled neutral lysis buffer (0.1M Na_2_EDTA·2H_2_O, 2.5 M NaCl, 1% Triton X-100, 10% DMSO, and 10mM Tris, pH 8.0) at 4℃ in the dark for 4 hours. In order to unwind the cellular DNA, the slides were submerged in the pre-cooled neutral electrophoresis buffer (90 mM Tris buffer, 90 mM boricacid, 2 mM Na_2_EDTA·2H_2_O, pH 8.5) at 4℃ for 20 minutes. Next, the damaged DNA fragments were electrophoresed for 30 minutes at a constant voltage of 20V·cm^−1^. After the slides being neutralized with 0.4 M Tris-HCl (pH 7.5) and left to air dry, samples were stained with the non-toxic DNA dye SYBR Green I at room temperature in the dark for 20 minutes and then examined under a fluorescence inverted microscope (Olympus IX71).

**Immunoprecipitation and DNA-RNA hybrid Immunoprecipitation (DR-IP)**

For assay C1orf109L interaction protein, HeLa cells were cultured on 10cm dishes and grown to confluence. When cells at 60-70% confluence, transfect pCMV-Flag-C1orf109L for Flag tagged C1orf109L express for 36 hours. Cells were washed once with phosphate-buffered saline (PBS) and harvested by scraping and centrifugation at 800 g for 5 minutes. The harvested cells were washed with PBS and lysed for 30 minutes on ice in the lysis buffer (25 mM Tris-HCl pH 8.0, 250 mM NaCl 1% Triton X-100) with Benzonase. Cell lysates were then spun down at 12,000 g for 20 min. The soluble fraction was collected, and the protein concentration was determined by Bradford assay. Next, 1 mg of extracted protein in lysis buffer was immunoprecipitated overnight with Flag-M2 affinity gel (Sigma) at 4℃. The immunoprecipitates were washed three times with lysis buffer. The beads were then eluted with 0.5 mg/ml of the corresponding antigenic peptide for 4 hours or directly boiled in SDS loading buffer.

For assay C1orf109L ubiquitination, breifly cells were lysed with lysis buffer (25 mM Tris-HCl pH 8.0, 250 mM NaCl, 1% Triton X-100, 1% SDS), and 100℃ for 5 minutes then ultrasonication with 30% 5 seconds stop 5 seconds for 5min. 12,000 g for 20 min remove supernatant into a new tube and ten times dilution. The protein concentration was determined by Bradford assay. Next, 1 mg of extracted protein was immunoprecipitated overnight with GFP anti-body and mix with protein A/G magnetic beads at 4℃. The immunoprecipitates were washed three times with lysis buffer and boiled in SDS loading buffer.

For DR-IP , briefly, cells were isolated nuclear, suspend cells with nucleic extract buffer (10 mM Hepes pH 7.9, 1.5 mM MgCl_2_, 0.34 M [sucrose](../../AppData/Local/youdao/dict/Application/7.5.2.0/resultui/dict/%3fkeyword=sucrose), 10% glycerol, 1 mM DTT, 10 mM KCl) with 1% protease inhibitor, Triton X-100 (1%) was added and the suspensions were incubated for 5 min on ice. Nuclei were collected in pellet by 1300 g for 5 minutes at 4℃, then lysed nuclei with lysis buffer (25 mM Tris-HCl pH 8.0, 250 mM NaCl, 1% Triton X-100) with or without 1μg/mL RNAse A for 30 minutes and ultrasonication until DNA fragment almost 200 bp. Finally 12,000 g for 20 minutes for supernatant, Supernatant was added S9.6 antibody for 4 hours. The [immunoprecipitation](../../AppData/Local/youdao/dict/Application/7.5.2.0/resultui/dict/%3fkeyword=immunoprecipitation)s were washed three times with lysis buffer and directly boiled in SDS loading buffer.

**Transcriptome analysis**

Total RNA was extracted from sorted cells using an RNeasy MicroKit (Qiagen) following the manufacturer’s instructions. The RNA concentration and quality were assessed during the Qubit RNA assay kit (Invitrogen). We used 300 ng of total RNA to prepare the TruSeqlibrary, for which we used the Illumina Low-Throughput TruSeq RNA Sample Preparation Kit protocol, resulting inbarcoded cDNA. Next, 50 ng of barcoded TruSeq products were used for Illu-minaRNA sequencing on an IlluminaHiSeq2000 sequencer to generate single-end 50 or 51-nucleotide reads according to the manufacturer’s protocol. The expression levels of each sample were normalized as Reads Per Kilobase Per Million (RPKM) by dividing the read count of each transcript model with its length and scaling the total per sample to one million.
